# Maximizing the Use of Ivermectin Transethosomal Cream in the Treatment of Scabies

**DOI:** 10.3390/pharmaceutics16081026

**Published:** 2024-08-01

**Authors:** Mohammad H. Alyami, Hamad S. Alyami, Asmaa M. Abdo, Shereen A. Sabry, Hanan M. El-Nahas, Margrit M. Ayoub

**Affiliations:** 1 Department of Pharmaceutics, College of Pharmacy, Najran University, Najran 66462, Saudi Arabia; 2 Department of Pharmaceutics, Faculty of Pharmacy, Zagazig University, Zagazig 44519, Egypt

**Keywords:** ivermectin, scabies, transethosomes, full factorial design, histopathological examination

## Abstract

In an effort to tackle the skin reactions frequently observed with topical application of ivermectin (IVM), a study was conducted to develop and optimize transethosomes (TESMs) loaded with IVM for scabies treatment. A three-factor, two-level (2^3^) full factorial design was employed. Soyabean phosphatidylcholine concentration (A), ethanol concentration (B) and Span 60 amount (C) were studied as independent factors, while entrapment efficiency (EE), particle size (PS), polydispersity index (PDI), zeta potential (ZP) and drug release after 6 h (Q6h) were characterized. The skin sensitivity of the optimized formulation was evaluated by skin irritation test and histopathological examination. The EE% ranged from 88.55 ± 0.576% to 94.13 ± 0.305%, PS was from 318.033 ± 45.61 nm to 561.400 ± 45.17 nm, PDI was from 0.328 ± 0.139 to 0.671 ± 0.103, ZP was from −54.13 ± 1.09 mV to −60.50 ± 2.34 mV and Q6h was from 66.20 ± 0.30% to 93.46 ± 0.86%. The IVM-loaded transethosomal cream showed lower skin irritation and a more intact epidermal layer with intact keratinocyte, compared to the marketed cream which showed severe destruction of the keratin layer. Therefore, patient compliance can be improved by encapsulating IVM within TESMs to minimize its skin reactions.

## 1. Introduction

Scabies is a contagious disease caused by an ectoparasite, *Sarcoptes scabiei* var. *hominis*, presenting with itchy skin lesions mainly around the navel, axillae, buttocks and the genitalia [1,2]. The worldwide prevalence of scabies has reached about 300 million individuals annually [3]. Intense itching (pruritus) and a pimple-like (papular) itch rash are the most common signs and symptoms of scabies [3]. The rash and itching may be limited to common sites such as the wrist, elbow, armpit, webbing between the fingers, nipple, penis, waist, belt-line and buttock or may affect much of the body [4]. The rash can also involve tiny blisters (vesicles), scales and the urge to scratch may be especially strong at night [5]. The disease is often transmitted by direct skin-to-skin contact [6]. Human scabies mites are capable of surviving in the environment (outside of the human body) for 24–36 h in normal room conditions (21 °C and 40–80% RH); during this time, they have the ability to infest [6,7]. Indirect transmission via clothing, bedding and other fomites may also be possible. It is difficult to control the outbreak of scabies. Therefore, it is considered an important public health problem in developed countries [8].

IVM has been used in developing countries for control of scabies and many other neglected tropical diseases (NTDs) at the community level [8]. IVM is a semisynthetic macrolide antibiotic isolated from *Streptomyces avermitilis*, and so belonging to the avermectines group [1]. It is used all over the world for various indications, including onchocerciasis, lymphatic filariasis, strongyloidiasis, human sarcoptic scabies, acarodermatitis and rosacea [9]. It stimulates the release of gamma aminobutyric acid (GABA) at the invertebrate nerve endings and muscle cells. Therefore, it interferes with the transmission of nervous impulses, causing an increase in membrane permeability, leading ultimately to neuromuscular paralysis and death of certain parasites [1]. The most frequently reported side effects of topical IVM have included skin burning sensation, skin irritation, pruritus [9], itching, redness, stinging of the skin, and dry skin [10]. These occurrences tend to diminish patient compliance [11].

Liposomes are phospholipid vesicles with one or more lipid bilayers surrounding an aqueous core. There are numerous studies about liposomes’ involvement in topical drug delivery [12,13]. After that, new nano-drug delivery systems were developed for topical application. Transferosomes (TFs) were introduced by Cevec and Blume; these are deformable liposomes composed of a phospholipid bilayer and surfactant [14]. The surfactant makes the vesicles more elastic and minimizes their rupture, particularly when applied to the skin. TFs have high deformability; they have the ability to pass through a narrow constriction (from 5 to 10 times less than their own diameter), which enhances the skin penetration of vesicles. In addition, the presence of surfactant molecules causes disruption of the lipid and protein packing within the *Stratum corneum* (SC) [15]. Many reports have shown that TFs were more effective than rigid liposomes. However, many studies have stated that TFs were unable to penetrate the lower layers of the SC. On the other hand, ethosomes (ETs) were introduced by Touito et al.; these are composed of PC and ethanol [16]. According to Maheshwari et al., the presence of ethanol increases the fluidity of SC lipids and interdigitates the lipid bilayer of vesicles, which improves the drug’s skin penetration [17]. After that, TESMs were introduced; these have the basic component of ETs in addition to surfactants [18]. So TESMs have the properties of both TFs and ETs [19].

The primary objective of this study is to develop TESMs that include IVM in order to reduce the occurrence of undesirable skin reactions caused by its topical application. For this purpose, different variables influencing vesicles’ characteristics were studied, employing a 2^3^ full factorial design, using Design Expert^®^ software, version 11. The optimized formulation underwent additional characterizations, including transmission electron microscopy (TEM), differential scanning calorimetry (DSC), Fourier transform infrared (FTIR) and a stability study. Additionally, the substance was incorporated into a cream and subjected to various physicochemical evaluations. A skin irritation test and histopathological study were also performed to assess the skin reactions of the prepared IVM-loaded transethosomal cream.

## 2. Materials and Methods

### 2.1. Materials

IVM was kindly supplied by Delta Pharma Co. (Cairo, Egypt). Soyabean phosphatidylcholine (SPC; Lipoid S-100) was kindly gifted by Lipoid, Switzerland. Span 60 was purchased from Sigma Aldrich Chemical Co. (St Louis, MO, USA). Ethyl alcohol, chloroform, potassium di-hydrogen orthophosphate, di-sodium hydrogen orthophosphate, stearic acid, glycerin, potassium hydroxide and sodium lauryl sulphate (SLS) were purchased from El-Naser pharmaceuticals Chemicals Co. (Cairo, Egypt). All other chemicals were of analytical grade and used as received.

### 2.2. Methods

#### 2.2.1. Experimental Design

In accord with the findings of preliminary studies, SPC concentration (A), which ranged from 2 and 4 (% *w*/*v*); ethanol concentration (B), which lay between 10 and 30 (% *v*/*v*); and Span 6o amount (C), which was either 15 or 35 (mg) were studied as independent variables. TESMs were prepared using a three-factor, two-level (2^3^) full factorial experimental design in order to prepare eight different formulations using Design-Expert^®^ software (version 11, Stat-Ease Inc, Minneapolis, MN, USA). The studied responses (dependent variables) were EE% (Y_1_), PS (Y_2_), PDI (Y_3_), ZP (Y_4_) and Q6h (Y_5_). Table 1 shows the factors examined, the two distinct levels of these factors, and the corresponding measured responses.

#### 2.2.2. Preparation of IVM-Loaded TESMs Formulations

The thin-film hydration method was followed to develop IVM-loaded TESMs, as described by Albash et al. [20]. First, SPC (200 or 400 mg), Span 60 (15 or 35 mg) and IVM (100 mg) were weighed and dissolved in chloroform in a clean dry pear-shaped flask. The organic solvents were slowly evaporated using a rotary evaporator (Basis Hei-VAP HL, Heidolph Instruments GmbH & Co. KG, Schwabach, Germany) under reduced pressure at 90 rpm. The rotating flask was kept in a water bath (Heizbad Hei-VAP, Heidolph Instruments GmbH & Co. KG, Schwabach, Germany) at 60 °C until a thin transparent film was formed. Then, the film was hydrated using 10 mL distilled water containing 10 or 30% ethanol at 60 °C, which is above the lipid phase transition temperature (TC) [21]. To ensure complete hydration of the film, glass beads were added and kept for 45 min. The vesicles’ dispersion was left overnight at 4 °C to obtain mature vesicles.

#### 2.2.3. Evaluation of IVM-Loaded TESMs Formulations

##### Measurement of Entrapment Efficiency (EE%)

The vesicular dispersion of the prepared formulations was centrifuged at 18,000 rpm for 1 h at 4 °C using a cooling centrifuge to separate the un-entrapped IVM. Then, the supernatant was withdrawn and suitably diluted to quantify the free IVM spectrophotometrically at λ_max_ (246 nm).

The EE% was calculated according to the following equation [22]:

EE% = [(TD − FD)/TD] ×100

where EE% is the percent of entrapment efficiency, TD is the amount of the total drug and FD is the amount of free drug.

##### Measurement of Particle Size (PS), Polydispersity Index (PDI) and Zeta Potential (ZP)

Measurements of PS, PDI and ZP of the prepared IVM-loaded TESMs were performed after being suitably diluted. A computerized Malvern Zetasizer Nano-ZS90 (Malvern Instruments Ltd., Malvern, UK) was used by selecting the relevant software option of the instrument. Each measurement was carried out in triplicate after equilibration at 25 °C for 2 min [23].

##### Measurement of the Cumulative Percentage of Drug Released after 6 h (Q6h)

An in vitro drug release study was performed to determine the percentage of IVM released from the fabricated TESMs formulations. The release of IVM from the prepared formulations was performed using the dialysis-bag method according to the method utilized by Abdallah et al. [24], with minor modifications. Briefly, TESMs dispersions equivalent to 10 mg of IVM were sealed in dialysis bags. Then, each dialysis bag containing a sample was immersed in a glass bottle containing 50 mL of phosphate buffer pH (5.5) containing 0.5% (*w*/*v*) of SLS. The bottles were shaken at 50 rpm and 32 ± 0.5 °C. Aliquots of 1 mL were taken from each bottle at different time intervals (0.5, 1, 2, 3, 4, and 6 h). Samples were suitably diluted and analyzed for their drug content spectrophotometrically at λ_max_ (246 nm) using phosphate buffer pH (5.5) containing 0.5% SLS as a blank. The dissolution medium was replaced with fresh medium to maintain the sink condition. Each sample was run in triplicate. Then, the standard error of the mean values was calculated.

##### Kinetic Study of Drug Release

To determine the in vitro release mechanism of all TESMs formulations, the release data were subjected to the following models: zero-order model (Q_t_ = K_o.t_), first-order model (log Q_t_ = log Q_o_ − K_.t_/2.303), Higuchi release model (Q_t_ = K_H.t_^0.5^), Korsmeyer–Peppas model (Q_t_/Q_∞_ = K_kp.t_^n^) and Hixson–Crowell model (Q_o_^1/3^ − Q_t_^1/3^ = K_s.t_). For each, Q_t_ is the amount of drug released at time (t); Q_o_ is the initial drug amount; Q_∞_ is the amount of drug released at time infinity (∞); K_o_, K, k_H_, K_kp_ and K_s_ are the release rate constants of the previous models, respectively; and n is the release exponent. The best-fitting model was determined by identifying the model with the highest correlation coefficient (R^2^). Additionally, the n value of the Korsmeyer–Peppas model confirmed the mechanism of drug release [25].

#### 2.2.4. Statistical Analysis, Optimization and Validation

Design-Expert^®^ software was used to evaluate the effects of the formulation variables on the investigated dependent variables. A one-way analysis of variance (ANOVA) test was adopted to analyze the obtained data to assess the model’s significance and prove the statistical analysis of the data based on the *p*-values [26,27]. The coefficient of determination (R^2^), predicted R^2^ and adjusted R^2^ were used to investigate the degree of fitness of the model to the experimental data [28]. The one-factor graphs were studied to determine whether there was a statistically significant correlation between the studied factors and the measured responses. To verify the selected model’s validity for various responses, linearity plots of observed-against-predicted values were also employed. The program software chooses the model which gives statistically accepted polynomial equations. These equations are utilized to demonstrate conclusions about each response after taking both the degree and sign of the calculated coefficient “A positive sign indicates synergism, whereas a negative sign indicates antagonism” [29]. The optimized formulation was chosen by utilizing a desirability function that focused on minimizing PS and PDI, while maximizing EE%, ZP (as an absolute value) and Q6h, as outlined in Table 1. Validity of the applied statistical models was verified by calculating the percentage of relative errors between the predicted values and the measured results by applying the following equation [26]:

% Relative error = [(predicted value − observed value)/predicted value] × 100

The optimized IVM-loaded TESMs formulation was evaluated by further characterization.

#### 2.2.5. Characterization of the Optimized IVM-Loaded TESMs Formulation

##### Transmission Electron Microscopy (TEM)

The morphology of the optimized IVM-loaded TESMs formulation was examined using a transmission electron microscope (JEOL JEM 1230; JEOL, Tokyo, Japan). The vesicular dispersion was applied onto a carbon-coated copper grid in the form of a thin film. Subsequently, the sample was subjected to staining with a 2% solution of phosphotungstic acid, followed by observation and photography [30].

##### Differential Scanning Calorimetry (DSC)

The thermal analysis of pure IVM, SPC, Span 60, freeze-dried powders of blank TESMs formulation (drug-free) and optimized IVM-loaded TESMs formulation was performed using a differential scanning calorimeter (DSC 60, Shimadzu Co., Kyoto, Japan). About 5 mg of each sample was added to an aluminum pan and heated to 200 °C at a heating rate equal to 10 °C/min under inert nitrogen flowing at a rate of 10 mL/min to prevent oxidation of the sample [31].

##### Fourier Transform Infrared Spectroscopy (FTIR)

Fourier transform infrared spectroscopy was conducted using the FTIR spectrometer (series 1600, Perkin-Elmer Corporation, Waltham, MA, USA). Samples of pure IVM, SPC, Span 60, blank TESMs formulation and optimized IVM-loaded TESMs formulation were mixed with potassium bromide and compressed in a hydraulic press to form pellets, and the pellets were then scanned in the range of 4000–400 cm^−1^ [32].

##### Stability Studies

The stability of the optimized IVM-loaded TESMs formulation was studied as a function of time and temperature regarding EE%, PS, PDI and ZP. The samples were stored in the air-tight colorless vials, kept away from light at 4 ± 1 °C and 25 ± 1 °C for 3 months. Samples were withdrawn at 1, 2 and 3 months and the results were compared with the initial measurements of the freshly prepared formulation [24]. The vesicles were examined for any change in their appearance and aggregation. Statistical analysis was performed by applying the two-way ANOVA test using GraphPad Prism, version 8.

#### 2.2.6. Preparation of IVM-Loaded Transethosomal Cream

In the subsequent study (in vivo experimental study), the formulation of the improved IVM-loaded TESMs was combined with a cream base to be used topically. The lipid phase was prepared by melting the stearic acid in a water bath at 80 °C. Potassium hydroxide flakes were dissolved in the aqueous phase (DW and glycerin), and a part of the aqueous phase was substituted by transethosomal dispersion containing the required quantity of IVM to prepare a cream containing 1% IVM. Then, the transethosomal part was added slowly as a part of the aqueous phase to the lipid phase (after stearic acid melting) with continuous triturating for 4 min in a water bath. The emulsion was cooled to room temperature to form a semisolid cream.

#### 2.2.7. Evaluation of the Prepared IVM-Loaded Transethosomal Cream

##### Physical Inspection

The homogeneity and texture of the developed IVM-loaded transethosomal cream were assessed by visual inspection and by pressing a small quantity of the prepared cream between the thumb and index finger [33].

##### Determination of pH Value

One gram of the prepared cream was dispersed in 25 mL of DW, and the pH was determined using a glass electrode of a digital pH meter. Determination of the pH was carried out in triplicate and the mean was taken [33].

##### Spreadability

This test aimed to investigate the spreadability of the developed cream and measure the diameter of spreading when applied to the affected area. Briefly, a definite amount of the cream was retained between two glass slides and a definite amount by weight (100 g) was applied on the upper slide for 1 min. The diameter of the spreading area was measured as an indicator of the spreadability [34].

##### Viscosity Measurement

The viscosity of the prepared IVM-loaded transethosomal cream was determined at room temperature using Viscometer-R with spindle no. 6. Measurement of the viscosity was carried out in triplicate and the mean was taken.

#### 2.2.8. In Vivo Experimental Study

##### Animals and Ethical Approval

Adult albino rats weighing 120~150 g each were utilized. The animals were obtained from the animal breeding center, Zagazig University, Egypt. They were kept for one week before starting the experiment in a 12 h light and 12 h dark cycle at room temperature with freely available water and food. The experiment was conducted according to the Institutional Animal Care and Use Committee (IACUC) guidelines of the Faculty of Pharmacy, Zagazig University (Approval number: ZU-IACUC/3/F/438/2022).

##### Skin Irritation Test

The skin irritation potential of the IVM-loaded TESMs cream was compared to that of the conventional, marketed IVM cream according to the protocol described by Castro et al. [11]. The number of animals was determined according to the 3R principles, and then the animals were randomly divided into three groups with 6 animals in each group [35], as follows: group I, TESMs-based cream without IVM (placebo cream); group II, marketed formulation (Iverzine^®^ cream containing 1% IVM, Unipharma, Egypt); and group III, IVM-loaded transethosomal cream (1% IVM). The hair was shaved from the back of all rats (6 cm^2^) 24 h before the application of the formulations. A definite amount of each formulation (0.25 g) was applied to the hair-free skin by uniform spreading. The rats were treated once a day for ten consecutive days. The rats were examined for erythema as a sign of skin irritation at 24 h after each application of the formulations. According to the degree of erythema (0 = no erythema; 1 = slight erythema (light pink); 2 = moderate erythema (dark pink); 3 = moderate to severe erythema (light red); and 4 = severe erythema (extreme redness)), the mean scores were recorded. Statistical analysis was performed by applying the two-way ANOVA test using GraphPad Prism, version 8. The primary irritation index (PII) was calculated according to the method described by Shah et al. [35] at 24 h, 48 h, 72 h and after the final application (10th day) to further assess the treatment sites as to the presence of irritation.

##### Histopathological Study

At the end of the skin irritation test, the animals were sacrificed, and the treated dorsal skin was removed and fixed in a 10% neutral-buffered formalin for 24 h, followed by washing and dehydration by increasing alcohol concentrations (50, 70, 80, 95 and 100%). Specimens were cleared in xylene and embedded in paraffin wax blocks. Then, specimens were sectioned by a sledge microtome (Rotary Leica RM2245; Leica Biosystems, Wetzlar, Germany). Finally, the specimens were deparaffinized and stained by hematoxylin–eosin staining (H–E stains) for histopathological examination using a light microscope (Axiostar Plus; ZEISS, Oberkochen, Germany) [36].

#### 2.2.9. Statistical Analysis

The observed responses of the formulations were analyzed using Design-Expert^®^ software, version 11 by applying a one-way analysis of variance (ANOVA) to determine the significance (*p*-value < 0.05). Another statistical analysis was conducted by applying two-way ANOVA using GraphPad Prism^®^, version 8 to determine the significance (*p*-value < 0.05).

## 3. Results and Discussion

### 3.1. Preliminary Studies for Preparation of IVM-Loaded TESMs

TESMs loaded with IVM were prepared using a conventional thin-film hydration method. This process was selected because the formation of thin films occurs over a surface area sufficient for full vesicle hydration, which enhances the EE% [37]. Preliminary trials were performed to select the surface-active agent (SAA) that produces TESMs with the highest EE%. It was noticed that TESMs prepared using Span 60 as a SAA exhibited higher EE% compared to those prepared with Tween 80). These findings are correlated to the edge activators’ HLB values. Edge activators with a low HLB value (HLB value of Span 60 = 4.7) produce TESMs with higher EE%, which results from the increased ratio of lipid volume in the prepared vesicles relative to the encapsulated aqueous volume [38]. Consequently, in this study, Span 60 was adopted as an EA to give flexibility to the TESMs’ membranes.

### 3.2. Evaluation of IVM-Loaded TESMs Formulations

#### 3.2.1. Influence of the Independent Variables on Entrapment Efficiency (EE%; Y_1_)

The EE% of the prepared TESMs formulations ranged from 88.55 ± 0.576% to 94.13 ± 0.305%, as indicated in Table 2. The linear model was the most suitable one when fitted to EE% data (*p*-value < 0.05), in which the difference between the adjusted and predicted R^2^ was small (less than 0.2), while the adequate precision was high, 21.0938 (greater than 4), as presented in Table 3. These determinations clarify the validity of the model and its ability to navigate the design space [39].

**Figure 1:**
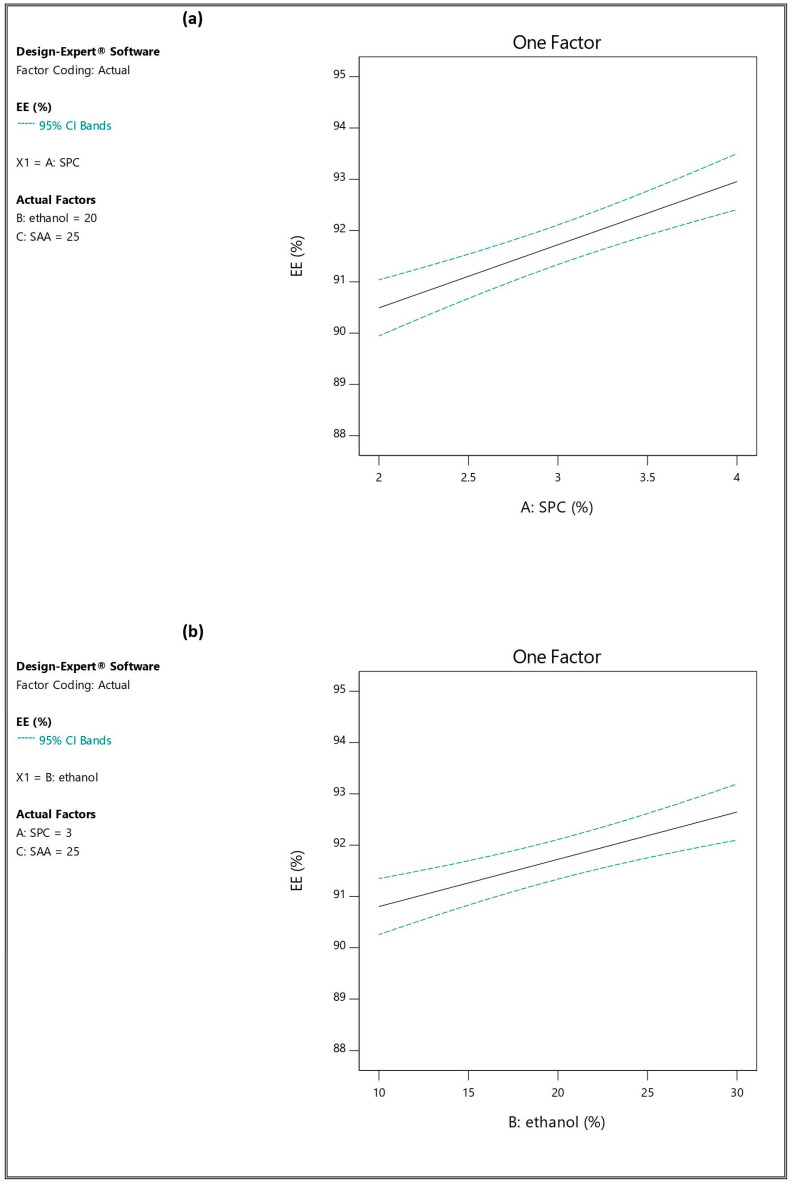

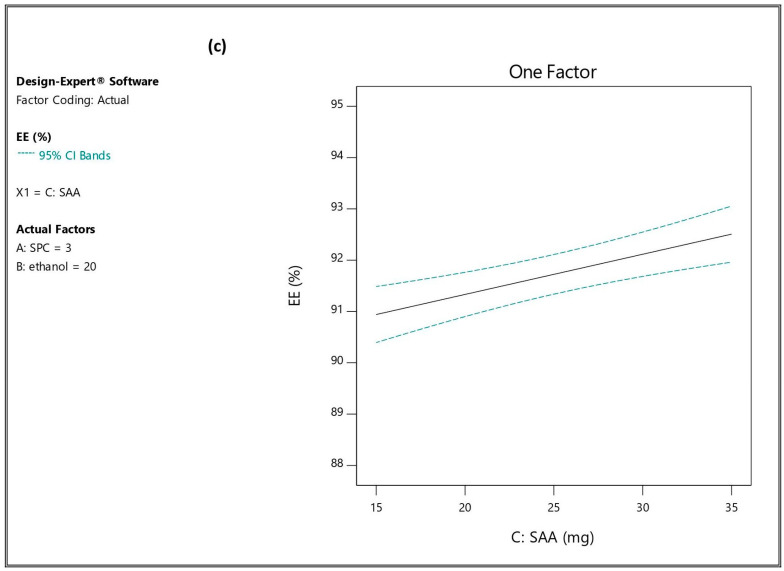
Influence of the independent factors on Y_1_ response (EE%): (**a**) effect of A at medium levels of B and C, (**b**) effect of B at medium levels of A and C, and (**c**) effect of C at medium levels of A and B.

ANOVA analysis revealed that SPC concentration (A), ethanol concentration (B) and edge activator (Span 60) amount (C) significantly affected EE% (*p*-value < 0.05). The effects of the studied factors on EE% are represented in Figure 1. When the SPC concentration increased from 2% [TESM1 (88.55 ± 0.576%), TESM2 (90.71 ± 0.520%), TESM5 (90.37 ± 0.416%) and TESM6 (92.34 ± 0.603%)] to 4% [TESM3 (91.10 ± 0.721%), TESM4 (93.40 ± 0.393%), TESM7 (93.19 ± 0.363%) and TESM8 (94.13 ± 0.305%)], there was a significant increase in the EE% of the corresponding formulations, respectively; similar results were reported by Garg et al. [19]. IVM belongs to class II in the biopharmaceutics classification system (BCS) and can also be classified as BCS class IV because it is a substrate for P-glycoprotein [40], so its hydrophobic nature is responsible for the interaction with the vesicle membrane, resulting in high EE% [41].

The co-solvent effect of ethanol may explain the significant increase in EE% observed when the concentration of ethanol was increased from 10% [(TESM1 (88.55 ± 0.576%), TESM3 (91.10 ± 0.721%), TESM5 (90.37 ± 0.416%) and TESM7 (93.19 ± 0.363%)] to 30% [(TESM2 (90.71 ± 0.520%), TESM4 (93.40 ± 0.393%), TESM6 (92.34 ± 0.603%) and TESM8 (94.13 ± 0.305%)], respectively [42]. The solubilization of the drug was enhanced by increasing the ethanol concentration, which subsequently improved the entrapment within both the inner hydroethanolic core and the lipid bilayers of the vesicle. This result is in agreement with Ahad et al. [42] and Peram et al. [43]. Figure S1a Supplementary Materials represented 3-D response surface plots showing the influence of A and B on EE%.

The amount of Span 60 also had a significant impact on EE%; formulations containing 35 mg [TESM5 (90.37 ± 0.416%), TESM6 (92.34 ± 0.603%), TESM7 (93.19 ± 0.363%) and TESM8 (94.13 ± 0.305%)] exhibited higher EE% than the corresponding ones containing 15 mg [TESM1 (88.55 ± 0.576%), TESM2 (90.71 ± 0.520%), TESM3 (91.10 ± 0.721%) and TESM4 (93.40 ± 0.393%)]. Similar results were reported by Abdallah et al. [24], who demonstrated that the EE% of silymarin increased with an increase in the edge activator concentration from 10 mg to 30 mg.

The fitted mathematical polynomial equation was derived from the studied design as follows:

EE = 91.72 + 1.23 A + 0.9212 B + 0.7837 C

#### 3.2.2. Influence of the Independent Variables on Particle Size (PS; Y_2_), Polydispersity Index (PDI; Y_3_) and Zeta Potential (ZP; Y_4_)

The prepared IVM-loaded TESMs showed PS varying between 318.033 ± 45.61 nm and 561.400 ± 45.17 nm, as shown in Table 2. The two-factor interaction (2-FI) model was the most suitable one when fitted to PS data (*p*-value < 0.05), in which the difference between the adjusted and predicted R^2^ was small (less than 0.2), while the adequate precision was high, 95.9126 (greater than 4), as presented in Table 3. These findings clarify the validity of the model and its ability to navigate the design space [27,38].

**Figure 2:**
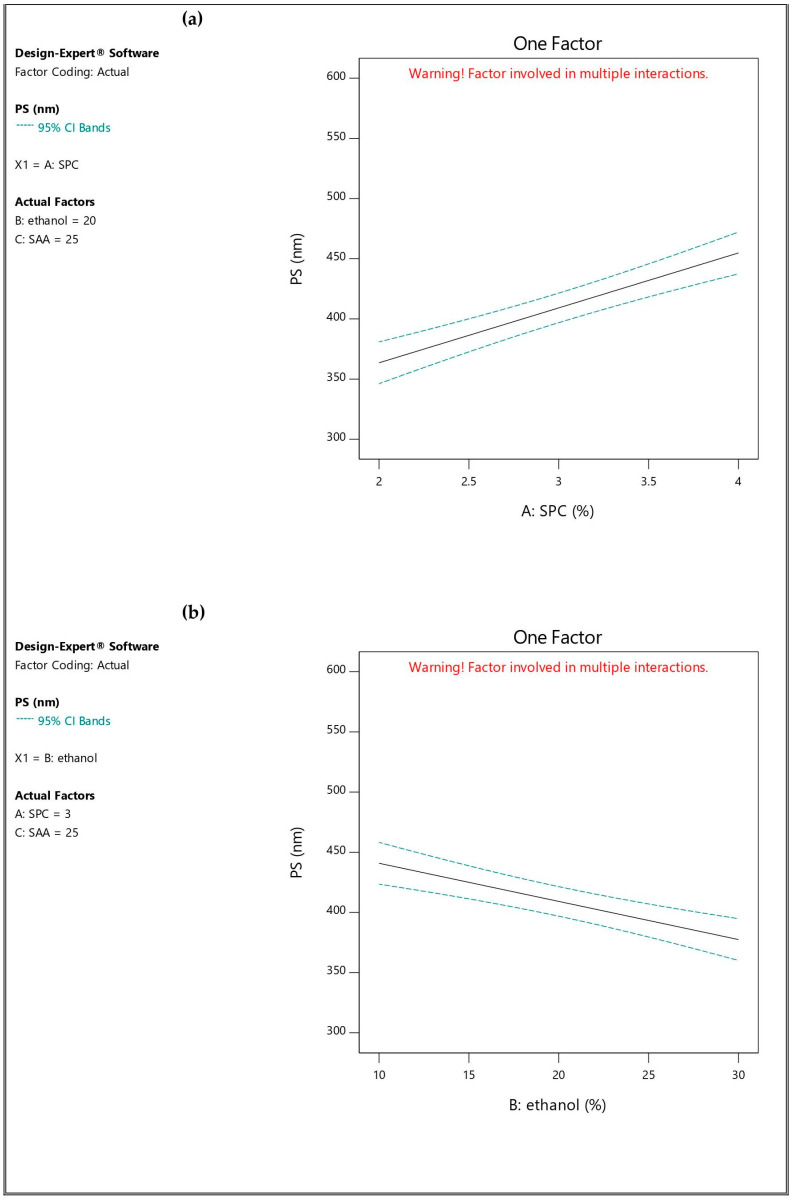

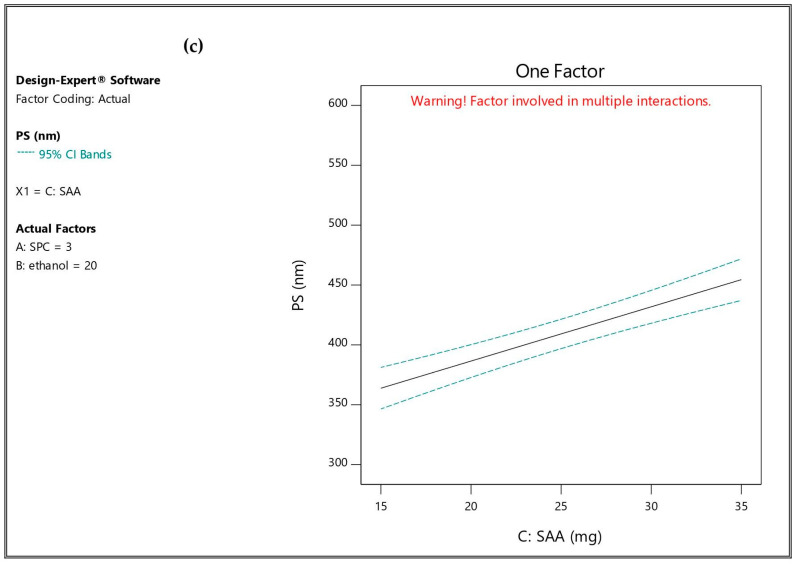
Influence of the independent factors on Y_2_ response (PS): (**a**) effect of A at medium levels of B and C, (**b**) effect of B at medium levels of A and C, and (**c**) effect of C at medium levels of A and B.

ANOVA analysis revealed that SPC concentration (A), ethanol concentration (B), edge activator (Span 60) amount (C), and the interaction between A and C significantly affected PS (*p*-value < 0.05). The effect of the independent factors on PS was graphically illustrated in Figure 2.

When the SPC concentration increased from 2% [TESM1 (363.267 ± 28.74 nm), TESM2 (318.033 ± 45.61 nm), TESM5 (415.200 ± 24.45 nm) and TESM6 (357.633 ± 31.81 nm)] to 4% [TESM3 (423.567 ± 35.38 nm), TESM4 (350.500 ± 43.25 nm), TESM7 (561.400 ± 45.17 nm) and TESM8 (483.733 ± 37.99 nm)], there was a significant increase in the PS of the corresponding formulations, respectively. Similar results were reported by Garg et al. [19] and Peram et al. [43]. The viscosity of the system was increased by increasing the amount of phospholipid, resulting in a reduced ability of the vesicles to be dispersed in the system; consequently, the PS will increase [44].

There was a significant reduction made in PS by increasing ethanol concentration from 10% to 30%, as clear in [TESM1 (363.267 ± 28.74 nm), TESM3 (423.567 ± 35.38 nm), TESM5 (415.200 ± 24.45 nm) and TESM7 (561.400 ± 45.17 nm)], versus [TESM2 (318.033 ± 45.61 nm), TESM4 (350.500 ± 43.25 nm), TESM6 (357.633 ± 31.81 nm) and TESM8 (483.733 ± 37.99 nm)], respectively. The negative charge provided by ethanol to the TESMs prevents aggregation of the vesicles due to steric repulsion, and hence the PS decreases. Similar results were reported by Peram et al. [43] and Ahad et al. [45].

The amount of Span 60 also had a significant impact on PS; formulations containing 35 mg [TESM5 (415.200 ± 24.45 nm), TESM6 (357.633 ± 31.81 nm), TESM7 (561.400 ± 45.17 nm) and TESM8 (483.733 ± 37.99 nm)] exhibited higher PS than the corresponding ones containing 15 mg [TESM1 (363.267 ± 28.74 nm), TESM2 (318.033 ± 45.61 nm), TESM3 (423.567 ± 35.38 nm) and TESM4 (350.500 ± 43.25 nm)]. Figure S1b–d Supplementary Materials represented 3-D response surface plots showing the influence of the independent factors on PS.

The fitted mathematical polynomial equation was derived from the studied design:

PS = 409.17 + 45.63 A − 31.69 B + 45.32 C − 5.99 AB + 22.44 AC − 2.12 BC

PDI is a parameter used to determine the homogeneity of the nanovesicles depending on their size distribution. Low values of PDI indicate a homogenous distribution, while high values specify a heterogenous distribution of vesicles [46]. The PDI of the prepared vesicles was in the range of 0.328 ± 0.139 to 0.671 ± 0.103, as shown in Table 2, indicating an acceptable size distribution for the prepared formulations [47]. Factorial analysis showed that the independent variables, SPC concentration (A), ethanol concentration (B), Span 60 amount (C) and their interactions, showed non-significant effects on PDI with *p*-values > 0.05, as represented in Table 3. Figure S1e–g Supplementary Materials represented 3-D response surface plots showing the influence of the independent factors on PDI.

**Figure 3:**
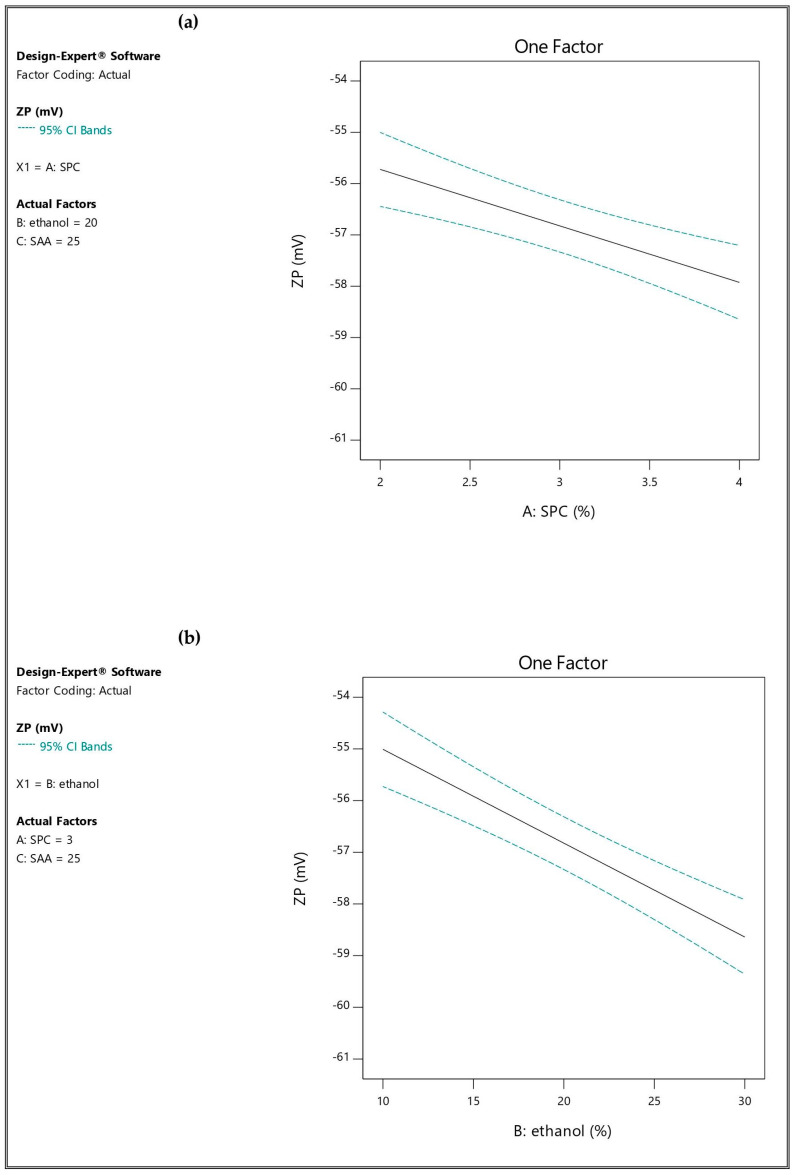

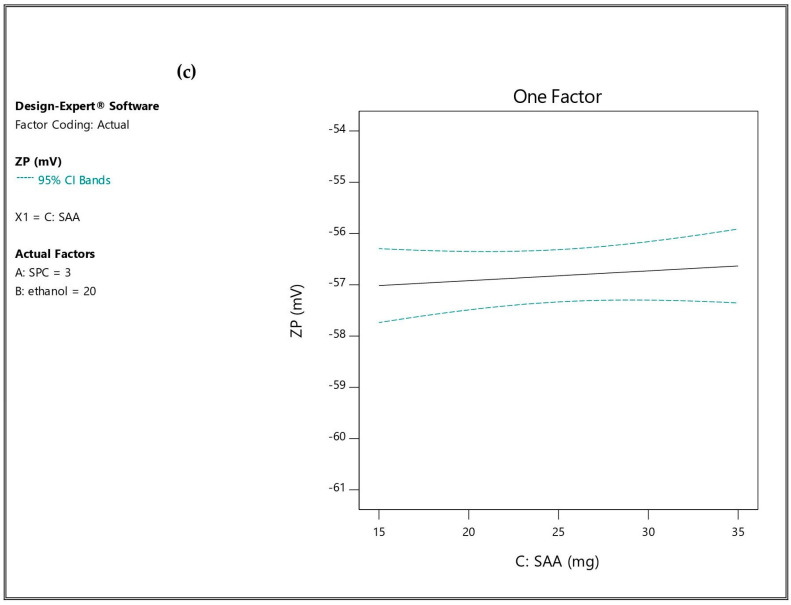
Influence of the independent factors on Y_4_ response (ZP): (**a**) effect of A at medium levels of B and C, (**b**) effect of B at medium levels of A and C, and (**c**) effect of C at medium levels of A and B.

ZP is correlated to the surface charge of the vesicles, which can influence the physical stability of the formulation and vesicle–skin interaction. Charged vesicles having a ZP of ≥│30│mV are less likely to aggregate because of electrostatic repulsion [48]. The ZP values of all prepared formulations were found to be in the range of −54.13 ± 1.09 mV to −60.50 ± 2.34 mV, as depicted in Table 2. The ANOVA results showed that SPC concentration (A) and ethanol concentration (B) had significant effects (*p*-value < 0.05) on ZP, while Span 60 amount (C) had a non-significant effect (*p*-value > 0.05). The linear model was the most suitable one when fitted to ZP values (*p*-value < 0.05), in which the difference between the adjusted and predicted R^2^ was small (less than 0.2), while the adequate precision was high, 16.9278 (greater than 4), as presented in Table 3. This clarifies the validity of the model and its ability to navigate the design space [39]. The effect of the independent factors on ZP is graphically illustrated in Figure 3.

When the SPC concentration increased from 2% [TESM1 (−54.20 ± 2.08 mV), TESM2 (−57.13 ± 0.12 mV), TESM5 (−54.13 ± 1.09 mV) and TESM6 (−57.43 ± 1.01 mV)] to 4% [TESM3 (−56.23 ± 1.30 mV), TESM4 (−60.50 ± 2.34 mV), TESM7 (−55.47 ± 0.51 mV) and TESM8 (−59.50 ± 0.73 mV)], the ZP was more negative in all corresponding formulations. This finding is in line with what Nasri et al. [49] found.

There was a significant increase effected in the negative charge of the ZP by increasing the ethanol concentration from 10% to 30%, as clear in [TESM1 (−54.20 ± 2.08 mV), TESM3 (−56.23 ± 1.30 mV), TESM5 (−54.13 ± 1.09 mV) and TESM7 (−55.47 ± 0.51 mV)] versus [TESM2 (−57.13 ± 0.12 mV), TESM4 (−60.50 ± 2.34 mV), TESM6 (−57.43 ± 1.01 mV) and TESM8 (−59.50 ± 0.73 mV)]. This result was also reported by Nasri et al. [49] and Kandil et al. [50]. Ethanol induces a surface negative charge in the polar head region of a phospholipid, which varies depending on its concentration. Electrostatic repulsions, as described by Jain et al. [51], cause delay in the growth of vesicle aggregates. Figure S1h Supplementary Materials represented 3-D response surface plots showing the influence of A and B on ZP%.

The fitted mathematical polynomial equation was derived from the studied design:

ZP = −56.82 − 1.10 A − 1.82 B + 0.1913 C

#### 3.2.3. Influence of the Independent Variables on Percentage of Drug Released after 6 h (Q6h; Y_5_)

**Figure 4:**
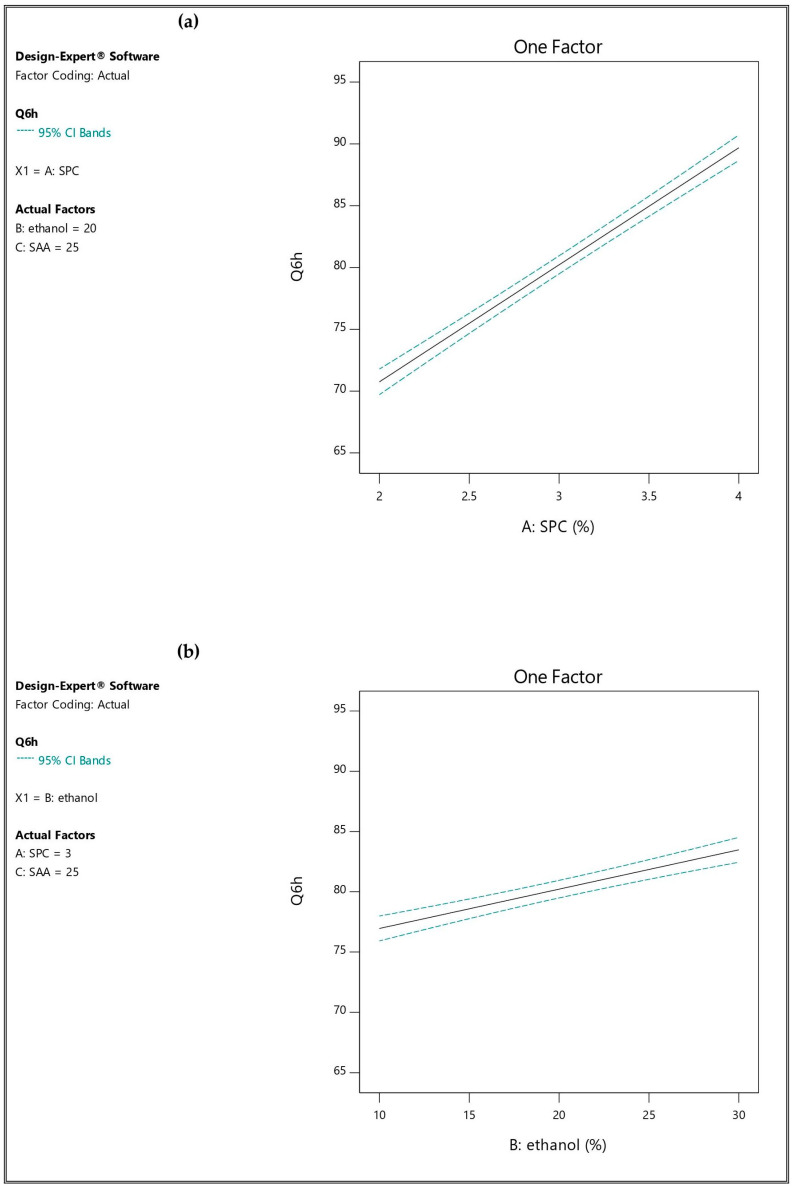

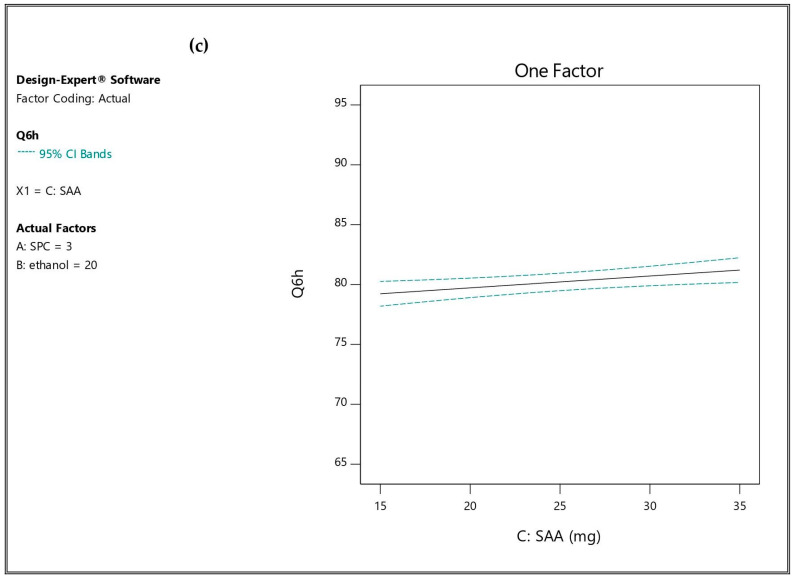
Influence of the independent factors on Y_5_ response (Q6h): (**a**) effect of A at medium levels of B and C, (**b**) effect of B at medium levels of A and C, and (**c**) effect of C at medium levels of A and B.

In vitro release profile is an essential tool used to predict the performance of the drug in the body. The release profiles for all the developed TESMs formulations are shown in Figure S2 in Supplementary Materials. The in vitro release studies of IVM from TESMs systems were studied at different time points over 6 h. The prepared formulations showed that Q6h varied between 66.20 ± 0.30% and 93.46 ± 0.86%, as shown in Table 2. The linear model was the most suitable one when fitted to Q6h data (*p*-value < 0.05), in which the difference between the adjusted and predicted R^2^ was small (less than 0.2), while the adequate precision was high, 52.147 (greater than 4), as presented in Table 3. These clarify the validity of the model and its ability to navigate the design space. The impact of the studied factors on Q6h is represented in Figure 4.

ANOVA analysis revealed that Q6h was significantly affected (*p*-value < 0.05) by SPC concentration (A), ethanol concentration (B) and Span 60 amount (C). When the SPC concentration increased from 2% [TESM1 (66.20 ± 0.30%), TESM2 (73.22 ± 1.70%), TESM5 (67.78 ± 1.01%) and TESM6 (75.79 ± 0.50%)] to 4% [TESM3 (86.03 ± 0.27%), TESM4 (91.46 ± 1.50%), TESM7 (87.80 ± 0.68%) and TESM8 (93.46 ± 0.86%)], there were significant increases in the Q6h of the corresponding formulations, respectively. Similar results were reported by Abdallah et al. [24].

Increasing the ethanol concentration from 10% to 30% resulted in a significant increase in Q6h, as clear in [TESM1 (66.20 ± 0.30%), TESM3 (86.03 ± 0.27%), TESM5 (67.78 ± 1.01%) and TESM7 (87.80 ± 0.68%)] versus [TESM2 (73.22 ± 1.70%), TESM4 (91.46 ± 1.50%), TESM6 (75.79 ± 0.50%) and TESM8 (93.46 ± 0.86%)]. It has been suggested that ethanol promotes drug diffusion from the vesicular membrane, as ethanol makes the membrane more flexible, while it also decreases the hydration layer around the vesicles, which facilitates drug release [30]. Figure S1i Supplementary Materials represented 3-D response surface plots showing the influence of A and B on Q6h%.

Variations in the amount of Span 60 also had a significant impact on Q6h; formulations containing 35 mg [TESM5 (67.78 ± 1.01%), TESM6 (75.79 ± 0.50%), TESM7 (87.80 ± 0.68%) and TESM8 (93.46 ± 0.86%)] exhibited higher Q6h than the corresponding ones containing 15 mg [TESM1 (66.20 ± 0.30%), TESM2 (73.22 ± 1.70%), TESM3 (86.03 ± 0.27%) and TESM4 (91.46 ± 1.50%)]. The formation of micelles within the bilayer is increased by increasing the edge activator concentration. Hence, drug permeation is enhanced [52]. This result is similar to that found by Abdallah et al. [30].

The fitted mathematical polynomial equation is derived from the studied design:

Q6h = 80.22 + 9.47 A + 3.26 B + 0.99 C

#### 3.2.4. Kinetic Study of Drug Release

The mechanism of IVM release from TESMs formulations was studied using in vitro release data from various mathematical models, as shown in Table 4. The Korsmeyer–Peppas model was the one that had the highest R^2^ value, making it the most suitable option for describing the process of IVM release. The n value of Korsmeyer–Peppas model was less than 0.5 for all formulations. This could indicate the degree of fit for drug release given a quasi-Fickian diffusion mechanism (case I transport) [53], whereas (n) is ≤0.5; this corresponds to a Fickian diffusion mechanism, while 0.5 < (n) < 1.0 corresponds to a non-Fickian mechanism (anomalous diffusion). The Fickian diffusion indicates that the release occurs by the diffusion of drug molecules due to the chemical potential gradient, while the non-Fickian diffusion indicates the combination of both diffusion and erosion-governed drug-release rate [54,55]. Many carrier systems follow a quasi-Fickian diffusion mechanism including solid lipid nanoparticles [56], polymeric micelles [57], proniosomes [58] and ethosomal gel [59]. Our results are in accordance with Farooq et al., who prepared variconazole-loaded transethosomal gel [60] and Asghar et al., who prepared transethosomal gel containing miconazole nitrate [61].

### 3.3. Optimization and Validation

**Figure 5:**
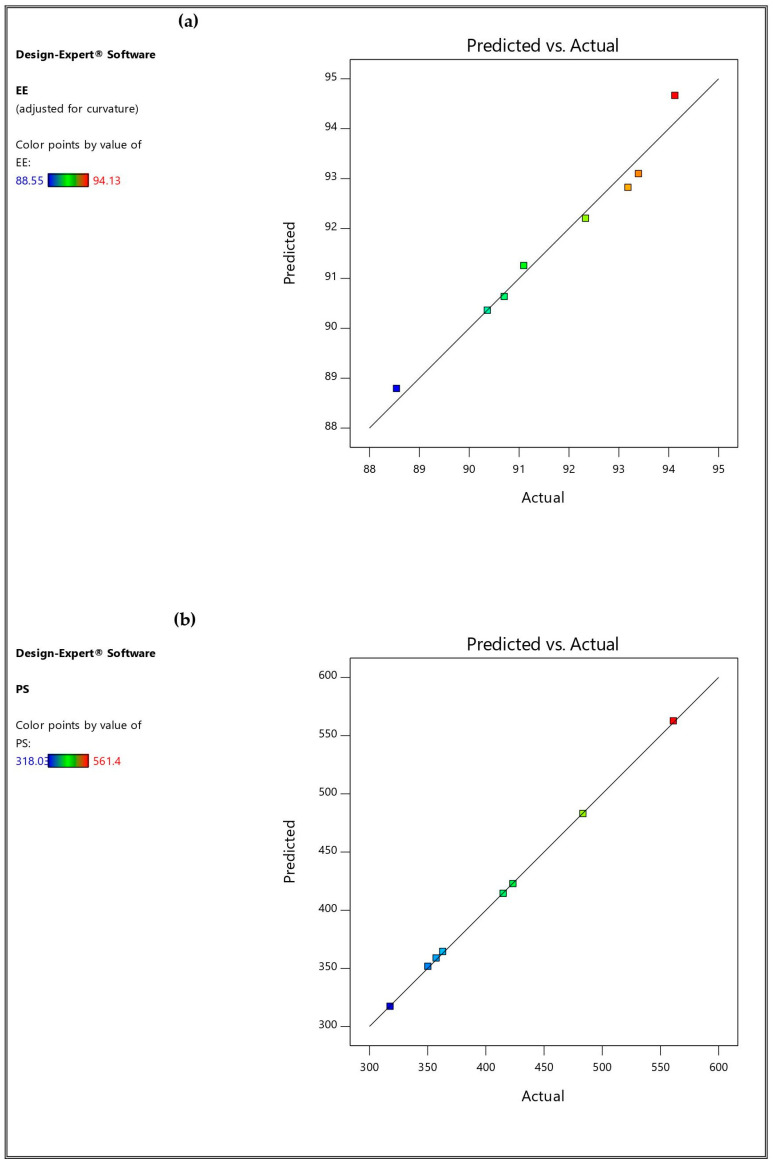

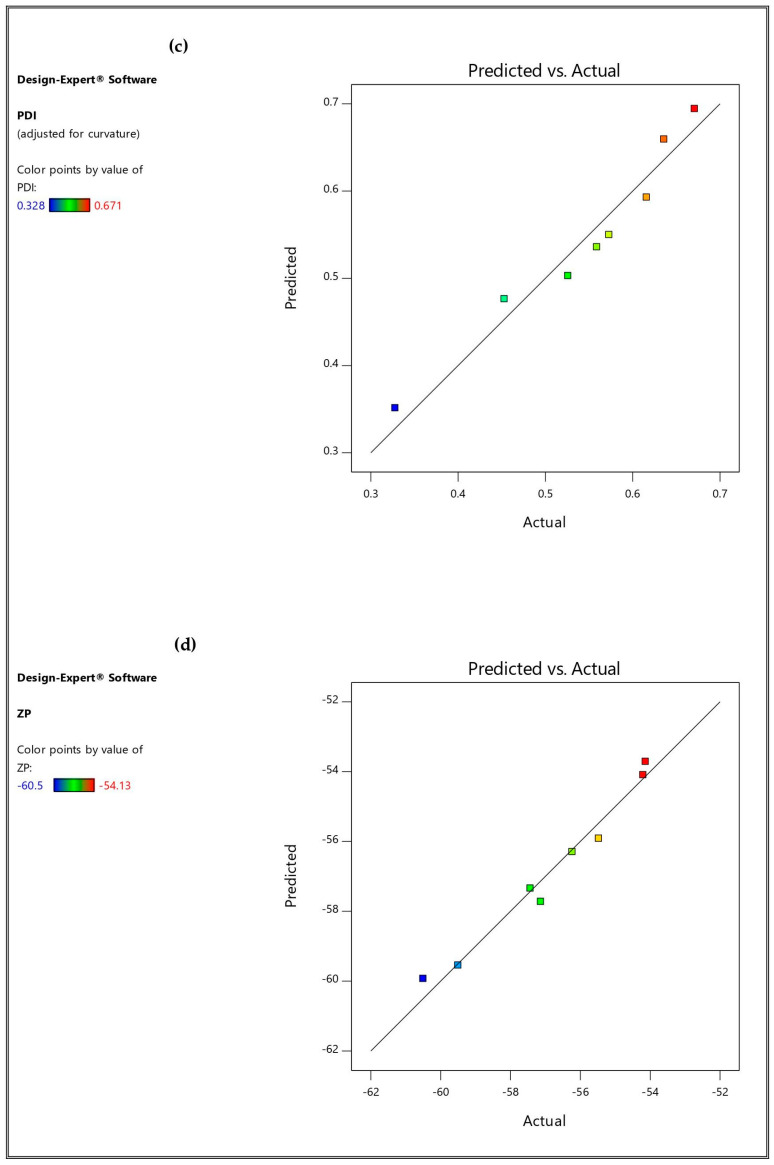

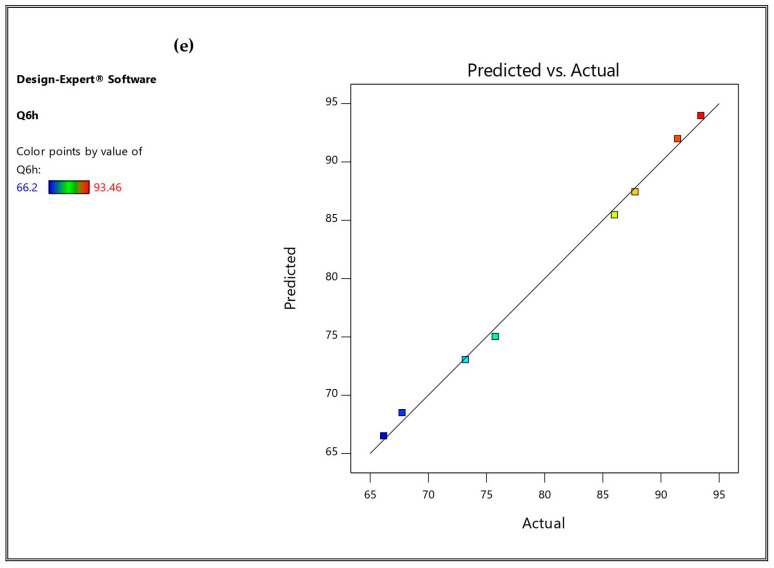
Linear correlation plots between actual and predicted values for all dependent variables: (**a**) EE%, entrapment efficiency; (**b**) PS, particle size; (**c**) PDI, polydispersity index; (**d**) ZP, zeta potential; (**e**) Q6h, cumulative amount of drug released after 6 h.

The optimization step was established to achieve the maximum EE%, ZP (as absolute value) and Q6h, and the minimum PS and PDI, as previously illustrated in Table 1. TESMs 4 was the one suggested by the design software as an optimized formulation which met the goal criteria and showed a high desirability value (0.892). The observed and predicted values of the optimized formulation were in a good agreement with% relative error values of less than 5% for EE%, PS, ZP and Q6h, as shown in Table 5. This could indicate the closeness of the observed and predicted values and the adequate ability of the optimization tools in the study. Hence, the validity, reliability and applicability of the models when used to appropriately describe the effects of studied factors on the measured responses were confirmed [62,63]. It was also obvious that there was a reasonably good agreement between the actual and predicted values for all responses, as illustrated in Figure 5.

### 3.4. Characterization of the Optimized IVM-Loaded TESMs

#### 3.4.1. Transmission Electron Microscopy (TEM)

**Figure 6:**
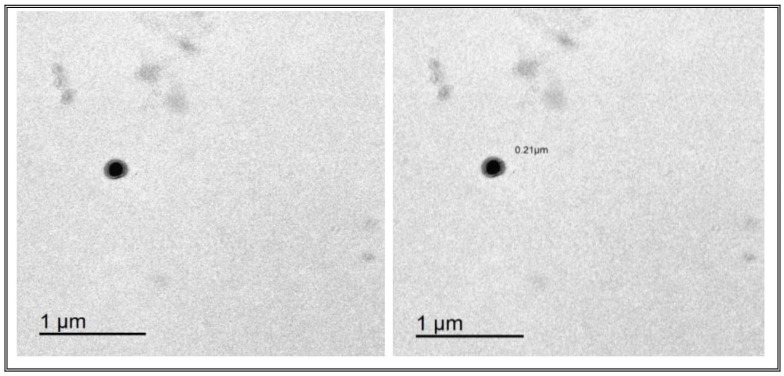
Transmission electron micrographs of the optimized TESMs formulation.

TEM analysis was used to study the external morphology of the optimized TESMs formulation. The analysis showed spherical bilayer vesicles with a uniform size distribution and no aggregate [64]. The PS of the vesicles as measured by the Zetasizer agreed well with TEM results, as shown in Figure 6.

#### 3.4.2. Differential Scanning Calorimetry (DSC)

**Figure 7:**
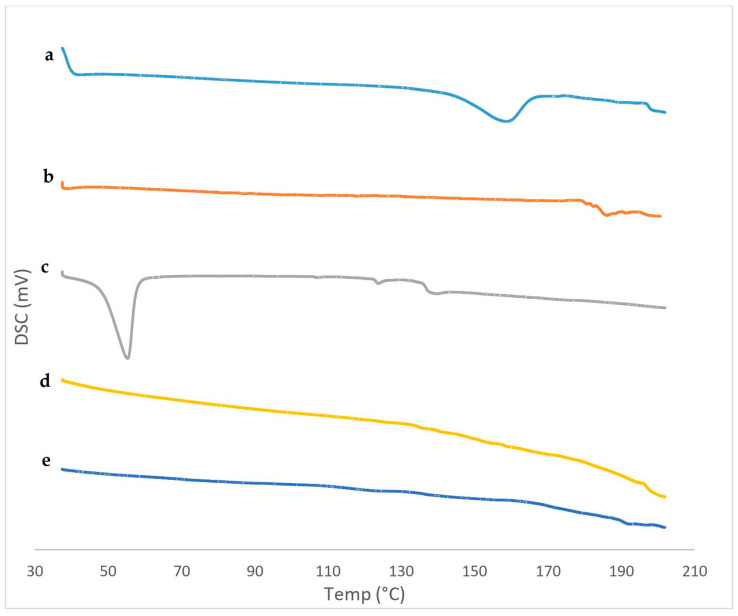
DSC thermograms of (**a**) pure IVM, (**b**) SPC, (**c**) Span 60, (**d**) blank TESMs formulation, and (**e**) optimized TESMs formulation.

DSC is an essential tool for detecting the physical state and thermal behavior of the drug. DSC thermograms of pure IVM, SPC, Span 60, the blank TESMs formulation and the optimized TESMs formulation are represented in Figure 7. The DSC thermogram of IVM depicted an endothermic melting peak at 158.69 °C, which revealed its crystallinity [65]. SPC demonstrated a characteristic peak at 187.42 °C corresponding to its melting temperature (transition from gel state to liquid-crystal state) [66,67,68], and Span 60 showed a characteristic endothermic peak at 54.2 °C [32]. The thermal behavior of the blank TESMs formulation demonstrated the possible interactions between SPC and Span 60, as seen by the disappearance of their characteristic peaks. The optimized TESMs formulation showed the absence of the characteristic peak of the drug, which indicates that the drug was converted into an amorphous form when incorporated into the nanovesicular system [30]; it also may indicate that the drug is completely entrapped inside the transethosomal vesicles due to interactions [26]. Formation of hydrogen bonds and Van der Waal’s attractive forces at the hydroxyl groups of IVM and both SPC and Span 60 are the most probable interactions. Formation of vesicles with favorable shape and good stability might be due to these interactions [20]. This finding was similar to the result reported by Peram et al. [43].

#### 3.4.3. Fourier Transform Infrared Spectroscopy (FTIR)

**Figure 8:**
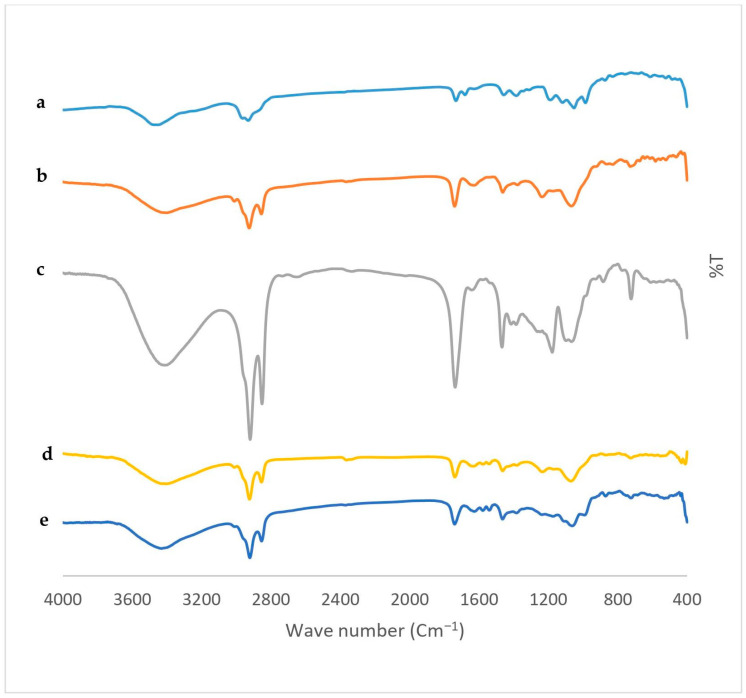
FTIR spectra of (**a**) pure IVM, (**b**) SPC, (**c**) Span 60, (**d**) blank TESMs formulation, and (**e**) optimized TESMs formulation.

FTIR was performed to illustrate the drug-excipient compatibility. FTIR spectra of pure IVM, SPC, Span 60, the blank TESMs formulation and the optimized TESMs formulation are shown in Figure 8. The FTIR spectrum of pure IVM displayed characteristic bands at 3450.99 cm^−1^, corresponding to the O-H stretching; 2931.27 cm^−1^, corresponding to the C-H stretching; 1732.73 cm^−1^ and 1690.66 cm^−1^, corresponding to the C=O stretching; 1458.96 cm^−1^, corresponding to the O-H bending; and 1394.64 cm^−1^, corresponding to the C-O stretching. SPC showed characteristic peaks at 2924.52 cm^−1^ and 2855.1 cm^−1^, corresponding to the stretching vibration absorption of CH_2_; 1740.44 cm^−1^, corresponding to the C=O stretching vibration of carboxylic acid; and 1462.74 cm^−1^, corresponding to the bending vibration of CH_3_ deformation [69]. The characteristic peaks of Span 60 appeared at 3413.39 cm^−1^, corresponding to the aliphatic O-H stretching; 2919.7 cm^−1^, corresponding to the C-H stretching; and 1737.55 cm^−1^, corresponding to the carbonylic C=O stretching of ester [70]. The FTIR spectrum of blank TESMs formulation exhibited the characteristic peaks of SPC and Span 60, along with reduced intensity, which may be due to the formation of the lipid bilayer. It was obvious that there was a non-significant change between FTIR spectra of the blank and optimized TESMs formulations, while there were no new peaks in the FTIR spectrum of the optimized TESMs formulation, confirming the absence of chemical interactions between the drug and the excipients. These findings were in agreement with results reported by Mazyed and Zakaria [26].

#### 3.4.4. Stability Study

**Figure 9:**
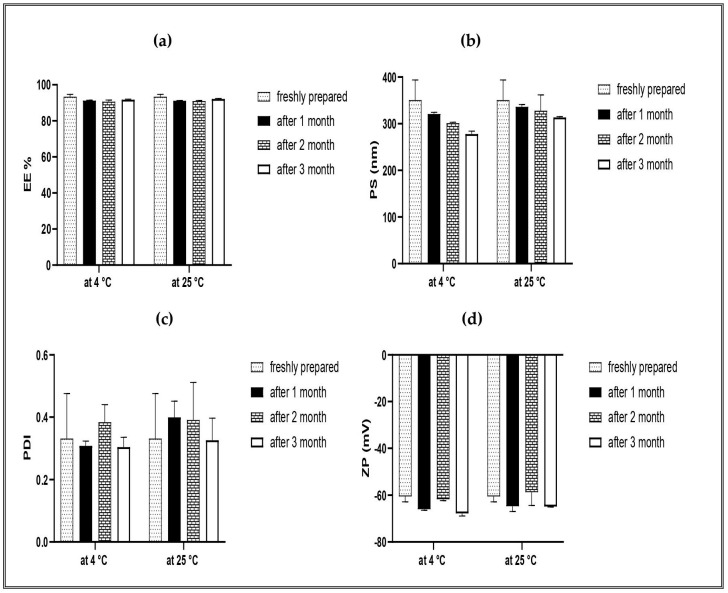
Outline of stability studies of optimized formulations for 1, 2 and 3 months at 4 ± 1 °C and 25 ± 1 °C in terms of (**a**) EE%, entrapment efficiency; (**b**) PS, particle size; (**c**) PDI, polydispersity index; and (**d**) ZP, zeta potential. Data were the mean ± SD.

One of the most important prerequisites for the application of nanovesicular formulations as a drug delivery system is the stability of the formulation. The results of stability studies for the optimized TESMs formulation are shown in Figure 9. Statistical analysis revealed that there was a non-significant change in the physical appearance, EE, PS, PDI and ZP of stored formulations at 4 ± 1 °C and 25 ± 1 °C, when compared to the fresh one (*p*-value > 0.05). Due to the high value of the negative charge for the ZP, an electrostatic repulsion was formed, and it prohibited any aggregation between vesicles, which led to an increased stability [71].

### 3.5. Evaluation of the Prepared IVM-Loaded Transethosomal Cream

The optimized IVM-loaded TESMs formulation was inserted into a cream base. Then, the prepared transethosomal cream was subjected to further characterization, with determinations of consistency, homogeneity, pH, spreadability and viscosity. The cream was found to be smooth and homogenous. The pH of the cream was found to be 6.73 ± 0.288; this was considered to be in the normal physiological pH range of the skin (4.5–7) [72], so it is suitable for topical application [24]. The distance travelled by the transethosomal cream when compressed between two slides was considered as an indicator for its spreadabilitiy [73]. The cream travelled a total distance of 2.93 ± 0.15 cm; this is in accord with the result reported by Chen et al. [33]. The cream’s viscosity was measured as 56,180 ± 242.5 cP; this result is similar to that reported by Chen et al. [33], indicating that it had suitable consistency for topical application.

### 3.6. In Vivo Experimental Study

#### 3.6.1. Skin Irritation Test

The outcomes of skin irritation test were based on the visual observation of erythema (redness). The repetitive topical applications of the marketed IVM cream (1% IVM) induced moderate to severe erythema. On the other hand, a negligible skin irritancy could be observed in rats treated with IVM-loaded TESMs cream (1% IVM) and no erythema was observed in rats treated with placebo cream. Statistical analysis revealed that there was a non-significant difference between he placebo-cream-treated group and the IVM-loaded transethosomal cream-treated group (*p*-value > 0.05), while there was a statistical significant difference between the IVM-loaded transethosomal cream-treated group and the marketed-IVM-cream-treated group (*p*-value < 0.05). Additionally, the PII for the marketed IVM cream treated group was higher than that observed for IVM-loaded transethosomal cream treated group, as shown in Table 6. Therefore, the skin irritation test indicated clearly that IVM-loaded transethosomal cream exhibited markedly less skin irritation than that observed for marketed IVM cream in a 10-day cumulative irritancy study.

#### 3.6.2. Histopathological Study

**Figure 10:**
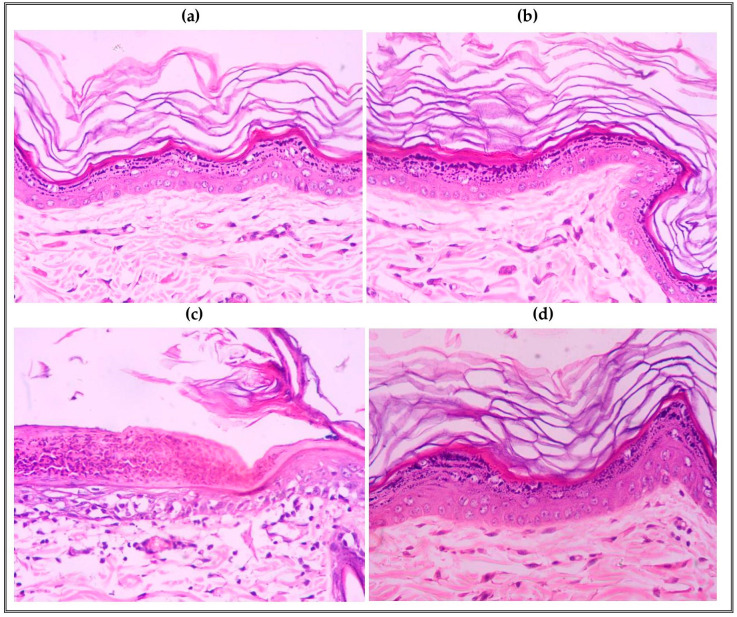
Photographs of H–E-stained sections: (**a**) control animal, (**b**) placebo cream group (I), (**c**) IVM marketed cream group (II), and (**d**) IVM-loaded transethosomal cream group (III).

Histopathological examination is an important tool used to study the inflammatory response of the skin tissue (Figure 10). It is clear from the light photographs that the placebo group (group I) showed a normal histological appearance of the skin, one approximately identical to that of the control animals. The group treated with marketed cream (group II) showed severe destruction of the keratin layer and degeneration of the epithelium cells, which showed small dark nuclei and lost cytoplasm; additionally, the underlying dermal layer showed a severe level of diffuse inflammatory cell infiltrates. These infiltrates are predominantly eosinophils, neutrophils and mononuclear leukocytes. Congested blood capillaries also were observed in the dermal layer. On the other hand, the group treated with IVM-loaded transethosomal cream (group III) showed an apparently intact thin epidermal layer with thick, intact keratinocyte, and a regular stratified squamous epithelium with regular and vesicular nuclei and an intact cytoplasm. Normal connective tissues were found in the dermis, along with a slight level of or lack of abnormal inflammatory cell infiltrates.

## 4. Conclusions

In this study, IVM-loaded TESMs were fabricated, characterized and optimized using a 2^3^ full factorial design and evaluated for their skin reactivity following topical application in a cream. The nano-sized, homogeneous vesicles have remarkable EE. The high negative surface charge prevented vesicle aggregation. The optimized formulation exhibited spherical vesicles when seen under TEM, with no signs of aggregation. Analysis using DSC and FTIR confirmed that the IVM was successfully encapsulated within the nanovesicles and demonstrated compatibility with the excipients. The three-month stability was good. Finally, the IVM-loaded transethosomal cream had less skin irritation than the marketed IVM cream; this was confirmed histopathologically. In conclusion, a novel strategy for treating scabies involves enclosing IVM within transethosomal vesicles, an intriguing tactic which can be used to reduce the skin reaction that IVM can cause.

## Figures and Tables

**Table 1:** Selected independent factors, dependent responses, and the criterion set for selecting the optimized formulation.

**Independent Factors**	**Factors Symbol**	**Unit**	**Factors Type**	**Factors Levels** **Low (−1) High (+1)**	
SPC concentration	A	% *w*/*v*	Numeric	2	4
Ethanol concentration	B	% *v*/*v*	Numeric	10	30
Span 60 amount	C	mg	Numeric	15	35
**Dependent responses**	**Responses symbol**	**Unit**	**Desirability constraints (goals)**		
Entrapment efficiency	Y_1_	%	Maximize		
Particle size	Y_2_	nm	Minimize		
Polydispersity index	Y_3_	---	Minimize		
Zeta potential	Y_4_	mV	Maximize (as absolute value)		
Cumulative amount of drug release after 6 h	Y_5_	%	Maximize		

**Table 2:** Eight experimental runs, as suggested by 2^3^ factorial design, and the observed values of the measured responses.

Formulation *	A (%)	B (%)	C (mg)	EE% (Y_1_)	PS (nm) (Y_2_)	PDI (Y_3_)	ZP (mV) (Y_4_)	Q6h (Y_5_)
**TESM1**	2	10	15	88.55 ± 0.576	363.267 ± 28.74	0.636 ± 0.096	−54.20 ± 2.08	66.20 ± 0.30
**TESM2**	2	30	15	90.71 ± 0.520	318.033 ± 45.61	0.559 ± 0.081	−57.13 ± 0.12	73.22 ± 1.70
**TESM3**	4	10	15	91.10 ± 0.721	423.567 ± 35.38	0.616 ± 0.113	−56.23 ± 1.30	86.03 ± 0.27
**TESM4**	4	30	15	93.40 ± 0.393	350.500 ± 43.25	0.328 ± 0.139	−60.50 ± 2.34	91.46 ± 1.50
**TESM5**	2	10	35	90.37 ± 0.416	415.200 ± 24.45	0.573 ± 0.257	−54.13 ± 1.09	67.78 ± 1.01
**TESM6**	2	30	35	92.34 ± 0.603	357.633 ± 31.81	0.671 ± 0.103	−57.43 ± 1.01	75.79 ± 0.50
**TESM7**	4	10	35	93.19 ± 0.363	561.400 ± 45.17	0.453 ± 0.164	−55.47 ± 0.51	87.80 ± 0.68
**TESM8**	4	30	35	94.13 ± 0.305	483.733 ± 37.99	0.526 ± 0.157	−59.50 ± 0.73	93.46 ± 0.86

Note: * All formulations contained 1% (*w*/*v*) of IVM. Abbreviations: A, SPC concentration (% *w*/*v*); B, ethanol concentration (% *v*/*v*); C, Span 60 amount (mg); EE%, entrapment efficiency; PS, particle size; PDI, polydispersity index; ZP, zeta potential; Q6h, cumulative amount of drug released after 6 h.

**Table 3:** Output data of 2^3^ full factorial design of IVM-loaded TESMs.

Measured Responses	Y_1_ (%)	Y_2_ (nm)	Y_3_	Y_4_ (mV)	Y_5_ (%)
**Suggested model**	linear	2-FI	2-FI	linear	linear
	*p*-value	*p*-value	*p*-value	*p*-value	*p*-value
**Model** **A** **B** **C** **AB** **AC** **BC**	0.0012 * 0.0009 * 0.0027 * 0.0049 * --- --- ---	0.024 * 0.0135 * 0.0194 * 0.0136 * 0.1018 0.0274 * 0.2727	0.4064 0.2202 0.4866 0.7299 0.4249 0.9522 0.2126	0.0015 * 0.0039 * 0.0006 * 0.3565 --- --- ---	<0.0001 * <0.0001 * 0.0002 * 0.0198 * --- --- ---
**Significant factors**	A, B, C	A, B, C, AC	---	A, B	A, B, C
**R^2^**	0.9746	0.9998	0.9498	0.9712	0.9973
**Adequate precision**	21.0938	95.9126	5.5760	16.9278	52.147
**Adjusted R^2^**	0.9556	0.9988	0.6484	0.9496	0.9952
**Predicted R^2^**	0.8986	0.9895	−2.2148	0.8848	0.9891

Abbreviations: Y_1_, entrapment efficiency (EE); Y_2_, particle size (PS); Y_3_, polydispersity index (PDI); Y_4_, zeta potential (ZP); Y_5_, cumulative amount of drug released after 6 h (Q6h); A, SPC concentration (% *w*/*v*); B, ethanol concentration (% *v*/*v*); C, Span 60 amount (mg). * Significant (*p*-value < 0.05).

**Table 4:** In vitro release kinetics data of the prepared TESMs formulation.

Release Model	R^2^							
	TESM1	TESM2	TESM3	TESM4	TESM5	TESM6	TESM7	TESM8
**Zero-order**	−0.6073	−0.5437	−0.6545	−0.5404	−0.6271	−0.5637	−0.1609	−0.7089
**First-order**	0.2456	0.4177	0.7239	0.8571	0.2252	0.4456	0.7882	0.9322
**Higuchi**	0.5096	0.5410	0.4751	0.5425	0.4919	0.5265	0.7311	0.4510
**Korsmeyer–Peppas**	0.9982	0.9959	0.9994	0.9992	0.9994	0.9992	0.9992	0.9971
**Hixson–Crowell**	0.0244	0.2121	0.4898	0.6218	0.0047	0.2545	0.6701	0.5899
**Best-fit model**	Korsmeyer–Peppas	Korsmeyer–Peppas	Korsmeyer–Peppas	Korsmeyer–Peppas	Korsmeyer–Peppas	Korsmeyer–Peppas	Korsmeyer–Peppas	Korsmeyer–Peppas
**n-value of Korsmeyer–Peppas**	0.417	0.453	0.438	0.430	0.370	0.381	0.383	0.379

**Table 5:** Predicted and observed values for the optimized formulation (TESMs4).

	Y_1_ (%)	Y_2_ (nm)	Y_3_	Y_4_ (mV)	Y_5_ (%)
**Predicted values**	93.093	351.467	0.351	−59.932	91.963
**Observed values**	93.4	350.5	0.328	−60.5	91.46
% **Relative error**	0.329	0.275	6.55	0.947	0.547

Abbreviations: Y_1_, entrapment efficiency (EE); Y_2_, particle size (PS); Y_3_, polydispersity index (PDI); Y_4_, zeta potential (ZP); Y_5_, cumulative amount of drug released after 6 h (Q6h).

**Table 6:** Scores of skin irritation test for rats treated with placebo cream, marketed IVM-cream and IVM-loaded transethosomal cream.

Animal No.	Placebo Cream				Marketed IVM-Cream				IVM-Loaded Transethosomal Cream			
	24 h	48 h	72 h	10 Days	24 h	48 h	72 h	10 Days	24 h	48 h	72 h	10 Days
**1**	0	0	0	0	0	1	1	1	0	0	0	0
**2**	0	0	0	0	1	1	2	2	0	1	1	1
**3**	0	0	0	0	1	1	1	1	1	1	1	1
**4**	0	0	0	0	1	2	2	1	0	0	0	0
**5**	0	0	0	0	2	2	2	3	0	0	0	0
**6**	0	0	0	0	2	2	2	2	1	1	1	1
PII*	0	0	0	0	1.17	1.5	1.67	1.67	0.33	0.5	0.5	0.5

Note: The sensitivity was scored as follows: 0 = no erythema; 1 = slight erythema (light pink); 2 = moderate erythema (dark pink); 3 = moderate to severe erythema (light red); 4 = severe erythema (extreme redness). PII*, primary irritation index.

## Data Availability

The data presented in this study are available in this article and the Supplementary Materials.

## Supplementary Materials

The following supporting information can be downloaded at: https://www.mdpi.com/article/10.3390/pharmaceutics16081026/s1, Figure S1: Array of 3-D response surface plots presenting the influence of the independent factors on the measured responses. (**a**) Effect of A and B on EE% at a medium level of C. (**b**) Effect of A and B on PS at a medium level of C. (**c**) Effect of A and C on PS at a medium level of B. (**d**) Effect of B and C on PS at a medium level of A. (**e**) Effect of A and B on PDI at a medium level of C. (**f**) Effect of A and C on PDI at a medium level of B. (**g**) Effect of B and C on PDI at a medium level of A. (**h**) Effect of A and B on ZP at a medium level of C. (**i**) Effect of A and B on Q6h at a medium level of C. Figure S2: In vitro drug release profiles of IVM-loaded TESMs formulations.
