# Supplementary material for: Maximizing the Use of Ivermectin Transethosomal Cream in the Treatment of Scabies
Source: Pharmaceutics. 2024 Aug 1;16(8):1026. doi: 10.3390/pharmaceutics16081026 (PMC11360360; doi:10.3390/pharmaceutics16081026)
Supplement: Supplementary file 1 [file pharmaceutics-16-01026-s001.zip › pharmaceutics-3095526-supplementary.pdf]

(a)

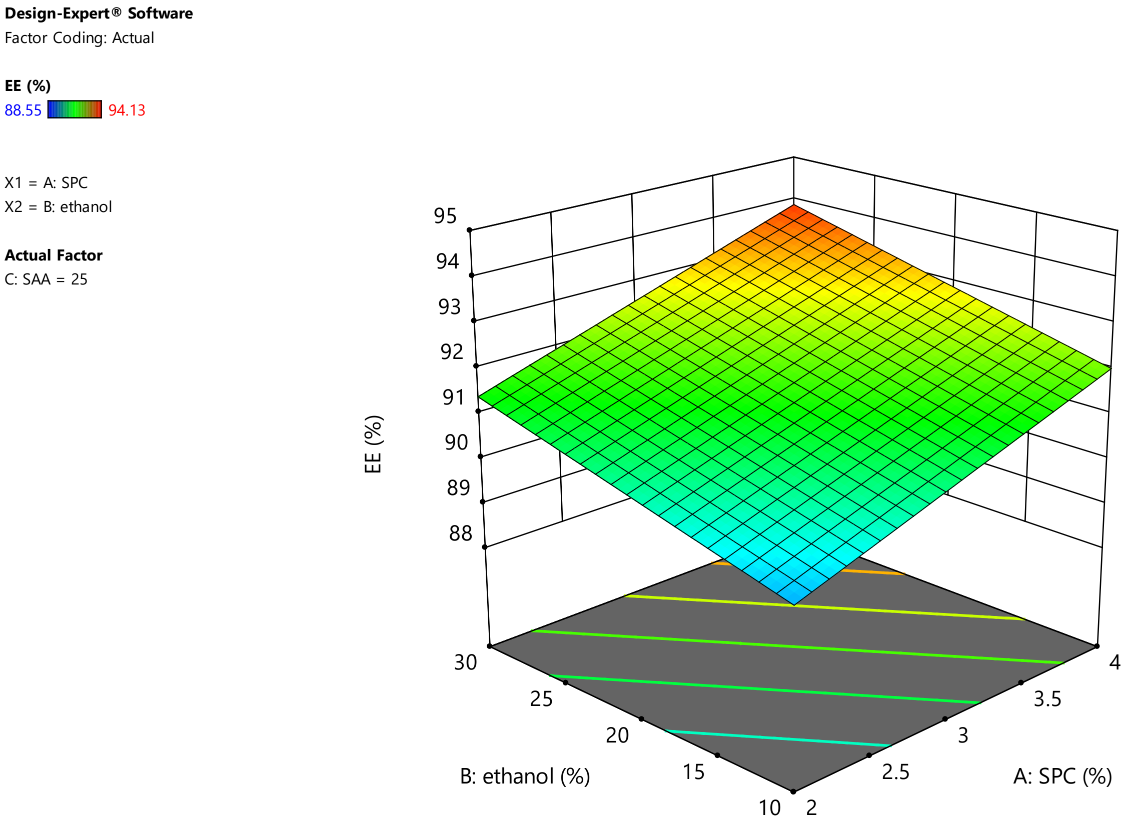
Design-Expert® Software
Factor Coding: Actual
EE (%)
88.55
94.13
X1 = A: SPC
X2 = B: ethanol
Actual Factor
C: SAA = 25
EE (%)
95
94
93
92
91
90
89
88
B: ethanol (%)
30
25
20
15
10
A: SPC (%)
2
2.5
3
3.5
4

(b)

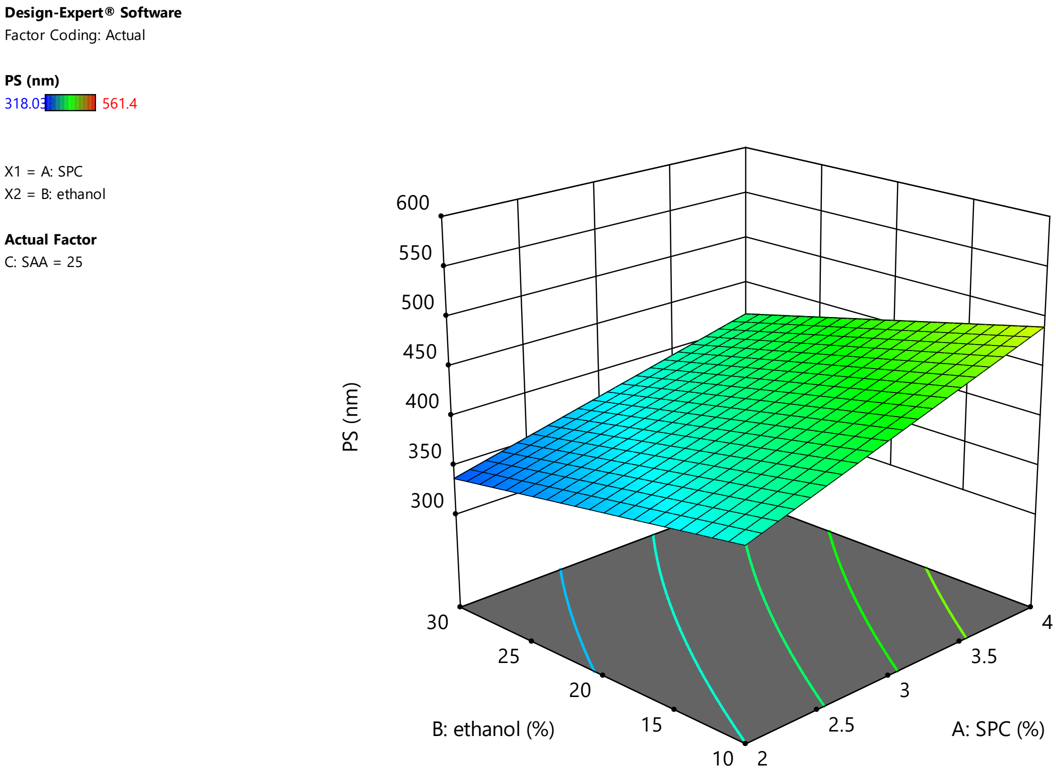
Design-Expert® Software
Factor Coding: Actual
PS (nm)
318.03
561.4
X1 = A: SPC
X2 = B: ethanol
Actual Factor
C: SAA = 25
PS (nm)
600
550
500
450
400
350
300
B: ethanol (%)
30
25
20
15
10
A: SPC (%)
2
2.5
3
3.5
4

(c)

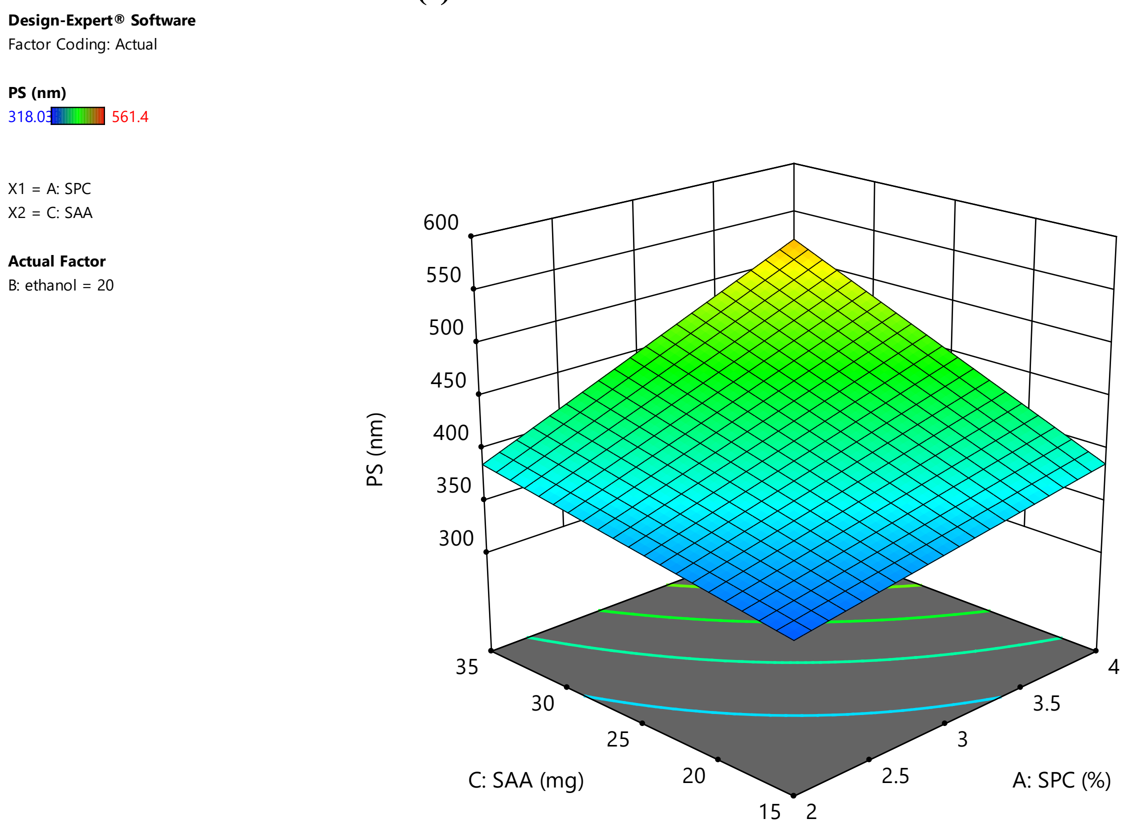
Design-Expert® Software
Factor Coding: Actual
PS (nm)
318.03
561.4
X1 = A: SPC
X2 = C: SAA
Actual Factor
B: ethanol = 20
600
550
500
450
400
350
300
PS (nm)
35
30
25
20
15
C: SAA (mg)
2
2.5
3
3.5
4
A: SPC (%)

(d)

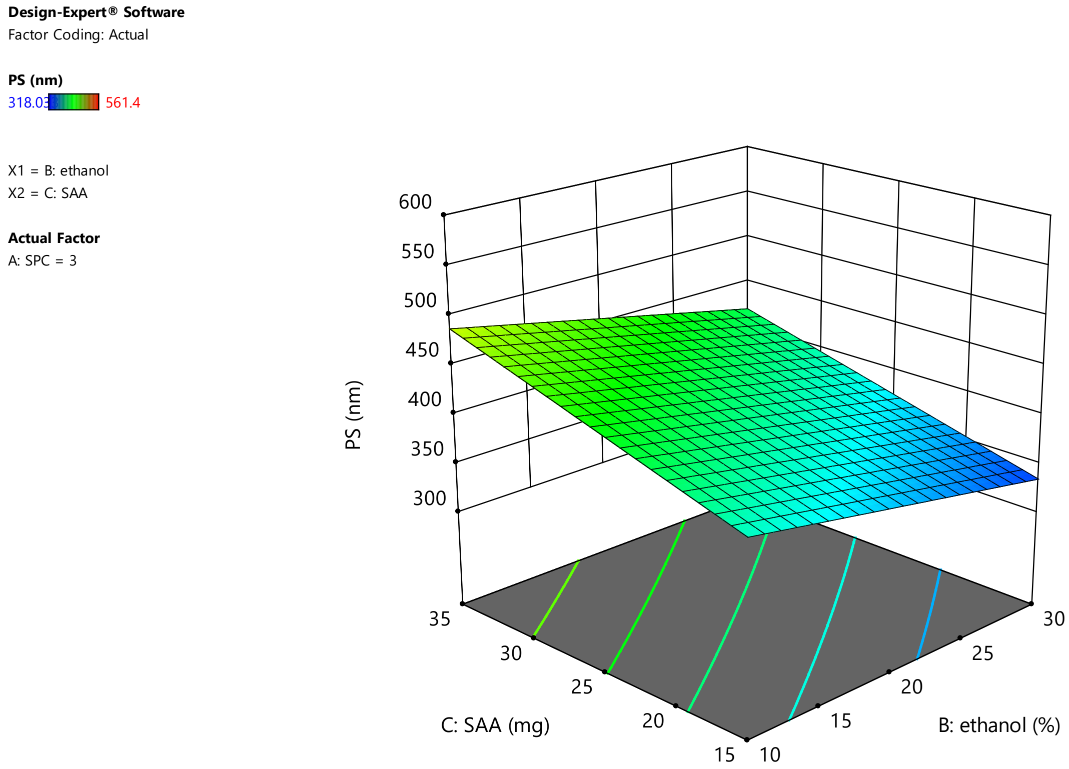
Design-Expert® Software
Factor Coding: Actual
PS (nm)
318.03
561.4
X1 = B: ethanol
X2 = C: SAA
Actual Factor
A: SPC = 3
600
550
500
450
400
350
300
PS (nm)
35
30
25
20
15
C: SAA (mg)
10
15
20
25
30
B: ethanol (%)

(e)

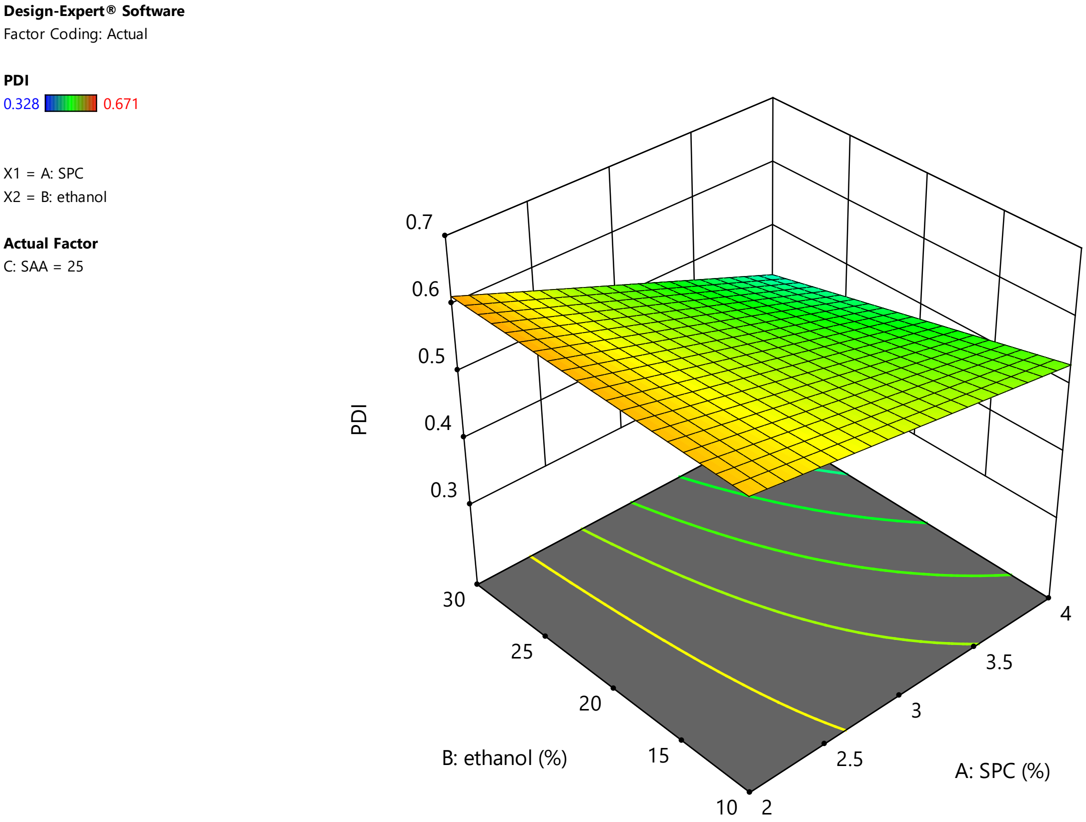

Design-Expert® Software
Factor Coding: Actual
PDI
0.328
0.671
X1 = A: SPC
X2 = B: ethanol
Actual Factor
C: SAA = 25
PDI
0.7
0.6
0.5
0.4
0.3
30
25
20
15
10
B: ethanol (%)
2
2.5
3
3.5
4
A: SPC (%)

(f)

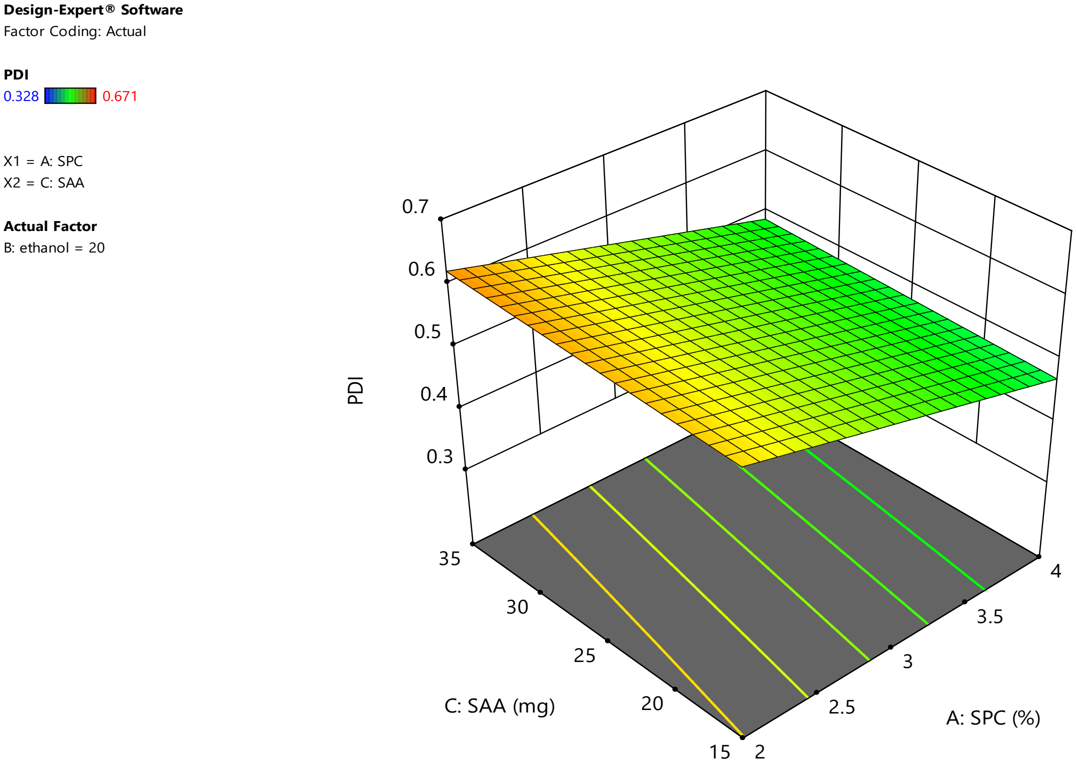

Design-Expert® Software
Factor Coding: Actual
PDI
0.328
0.671
X1 = A: SPC
X2 = C: SAA
Actual Factor
B: ethanol = 20
PDI
0.7
0.6
0.5
0.4
0.3
35
30
25
20
15
C: SAA (mg)
2
2.5
3
3.5
4
A: SPC (%)

(g)

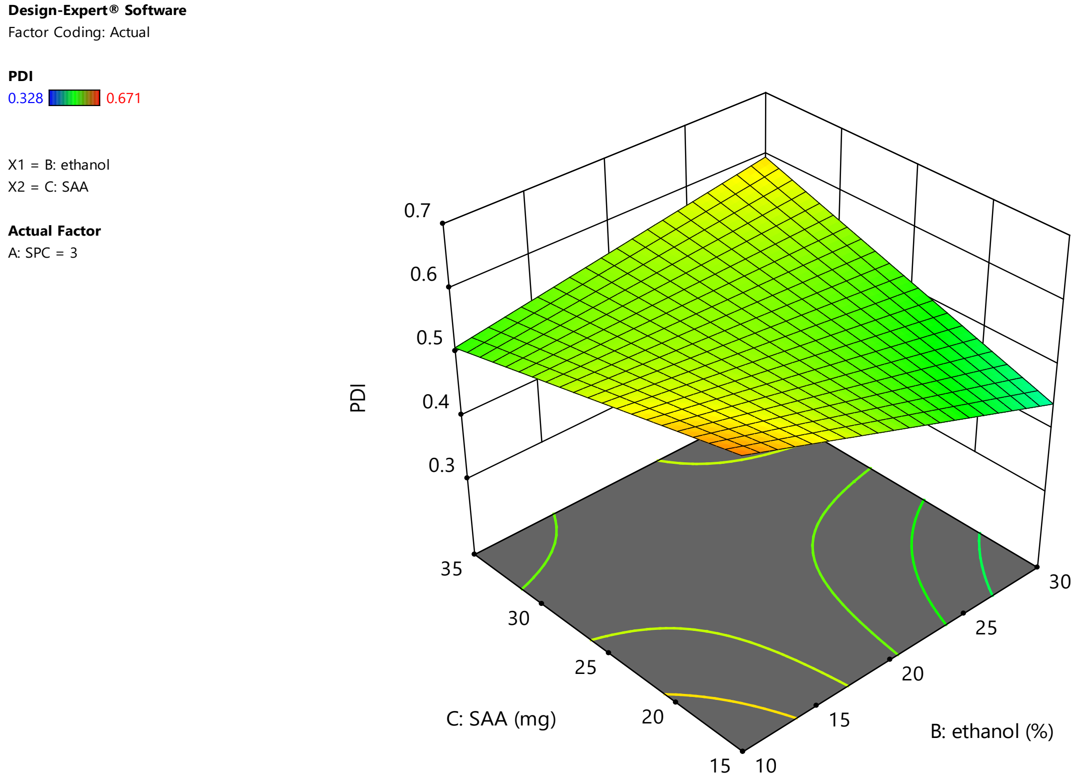
Design-Expert® Software
Factor Coding: Actual
PDI
0.328
0.671
X1 = B: ethanol
X2 = C: SAA
Actual Factor
A: SPC = 3
0.7
0.6
0.5
0.4
0.3
PDI
35
30
25
20
15
C: SAA (mg)
10
15
20
25
30
B: ethanol (%)

(h)

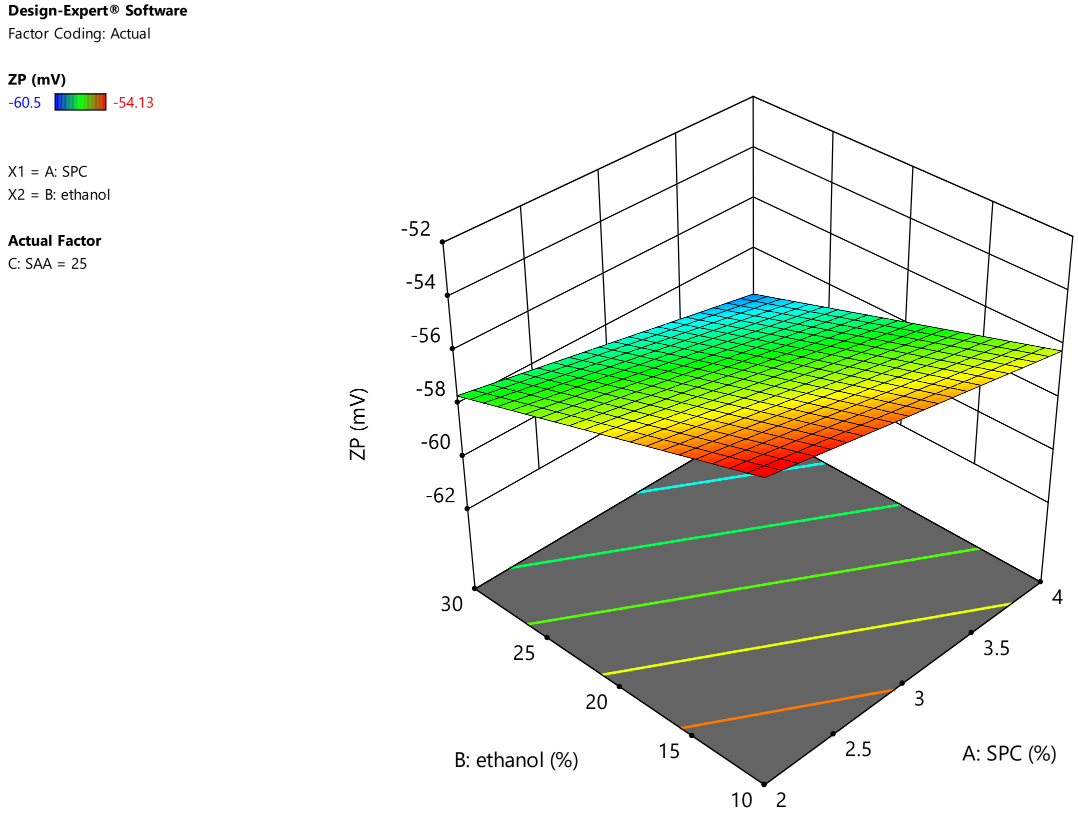
Design-Expert® Software
Factor Coding: Actual
ZP (mV)
-60.5
-54.13
X1 = A: SPC
X2 = B: ethanol
Actual Factor
C: SAA = 25
-52
-54
-56
-58
-60
-62
ZP (mV)
30
25
20
15
10
B: ethanol (%)
2
2.5
3
3.5
4
A: SPC (%)

(i)

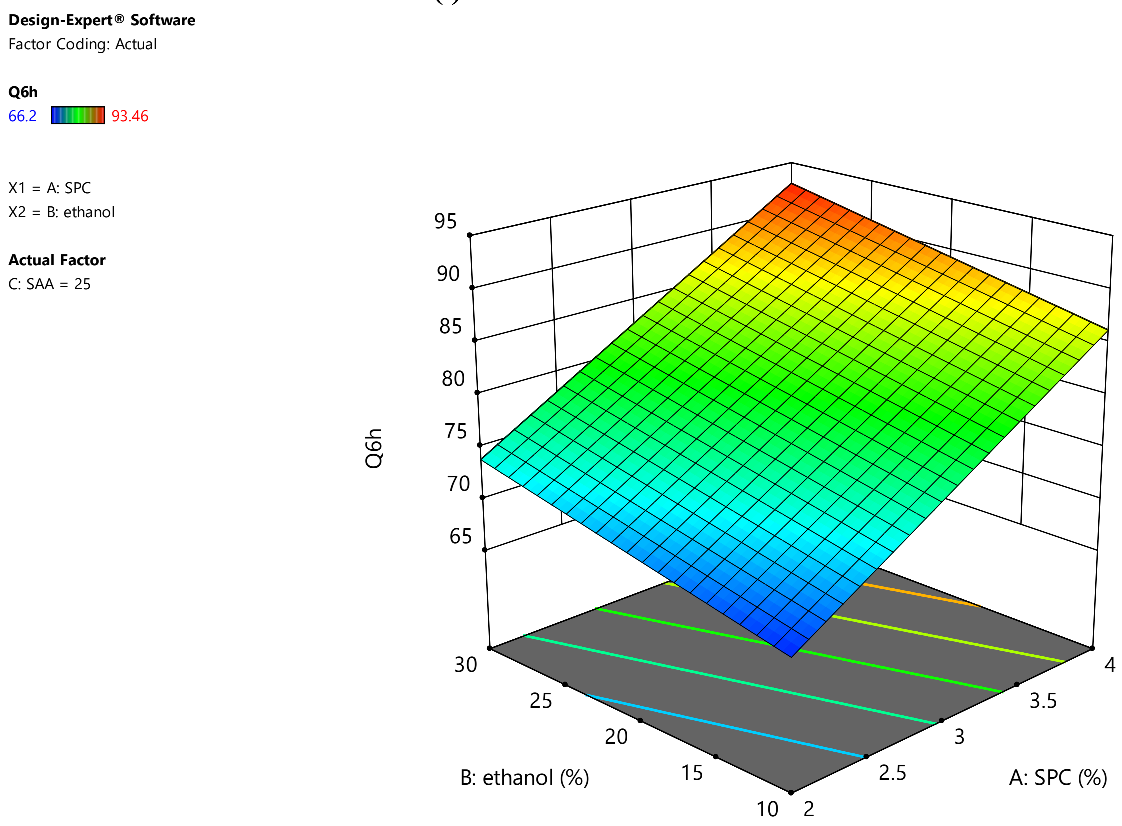

Figure S1. 3-D response surface plots presenting the influence of the independent factors on the measured responses. **(a)** effect of A and B on EE% at medium level of C. **(b)** effect of A and B on PS at medium level of C. **(c)** effect of A and C on PS at medium level of B. **(d)** effect of B and C on PS at medium level of A. **(e)** effect of A and B on PDI at medium level of C. **(f)** effect of A and C on PDI at medium level of B, **(g)** effect of B and C on PDI at medium level of A. **(h)** effect of A and B on ZP at medium level of C. **(i)** effect of A and B on Q6h at medium level of C.

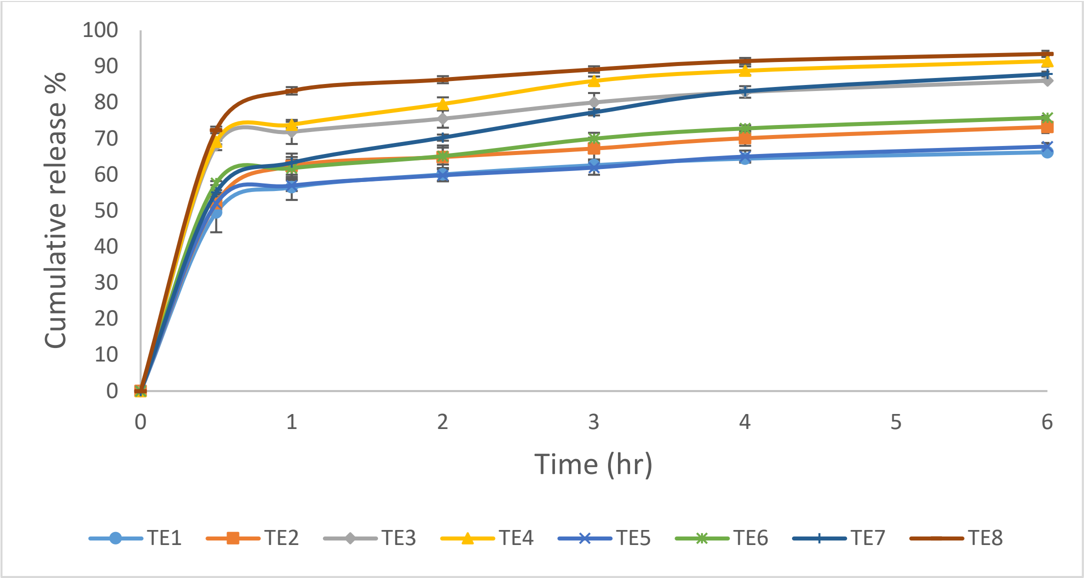

Figure S2. In-vitro drug release profiles of IVM-loaded transethosomal formulations.
